# G3BP1, G3BP2 and CAPRIN1 Are Required for Translation of Interferon Stimulated mRNAs and Are Targeted by a Dengue Virus Non-coding RNA

**DOI:** 10.1371/journal.ppat.1004242

**Published:** 2014-07-03

**Authors:** Katell Bidet, Dhivya Dadlani, Mariano A. Garcia-Blanco

**Affiliations:** 1 Program in Emerging Infectious Diseases, Duke-NUS Graduate Medical School, Singapore; 2 NUS Graduate School for Integrative Sciences and Engineering, National University of Singapore, Singapore; 3 Center for RNA Biology, Duke University Medical Center, Durham, North Carolina, United States of America; 4 Department of Molecular Genetics and Microbiology, Duke University Medical Center, Durham, North Carolina, United States of America; 5 Department of Medicine, Duke University Medical Center, Durham, North Carolina, United States of America; Harvard Medical School, United States of America

## Abstract

Viral RNA-host protein interactions are critical for replication of flaviviruses, a genus of positive-strand RNA viruses comprising major vector-borne human pathogens including dengue viruses (DENV). We examined three conserved host RNA-binding proteins (RBPs) G3BP1, G3BP2 and CAPRIN1 in dengue virus (DENV-2) infection and found them to be novel regulators of the interferon (IFN) response against DENV-2. The three RBPs were required for the accumulation of the protein products of several interferon stimulated genes (ISGs), and for efficient translation of PKR and IFITM2 mRNAs. This identifies G3BP1, G3BP2 and CAPRIN1 as novel regulators of the antiviral state. Their antiviral activity was antagonized by the abundant DENV-2 non-coding subgenomic flaviviral RNA (sfRNA), which bound to G3BP1, G3BP2 and CAPRIN1, inhibited their activity and lead to profound inhibition of ISG mRNA translation. This work describes a new and unexpected level of regulation for interferon stimulated gene expression and presents the first mechanism of action for an sfRNA as a molecular sponge of anti-viral effectors in human cells.

## Author Summary

Dengue virus is the most prevalent arbovirus in the world and an increasingly significant public health problem. Development of vaccines and therapeutics has been slowed by poor understanding of viral pathogenesis. Especially, how the virus subverts the host interferon response, a powerful branch of the innate immune system remains the subject of debate and great interest. Dengue virus produces large quantities of a non-coding, highly structured viral RNA, termed sfRNA, whose function in viral replication is elusive but has been linked in related viruses to inhibition of the interferon response. Nonetheless the mechanisms involved are yet to be characterized. Here, we show that dengue virus 2 sfRNA targets and antagonizes a set of host RNA-binding proteins G3BP1, G3BP2 and CAPRIN1, to interfere with translation of antiviral interferon-stimulated mRNAs. This activity impairs establishment of the antiviral state, allowing the virus to replicate and evade the interferon response. While this particular mechanism was not conserved among other flaviviruses, we believe it is highly relevant for dengue virus 2 replication and pathogenesis. Taken together, our results highlight both new layers of complexity in the regulation of the innate immune response, as well as the diversity of strategies flaviviruses employ to counteract it.

## Introduction

The critical roles of type I interferon (IFN) in detecting and clearing a wide range of viral infections have been well established [1]. IFNs are produced and released into the extracellular space by virtually all cell types upon recognition of pathogen-associated molecular patterns. Secreted IFNs act on the producing and neighboring cells to induce transcriptional activation of hundreds of antiviral IFN-stimulated genes (ISGs), establishing an antiviral state that rapidly targets viruses at various steps of their life cycle. While the transcriptional regulation of ISGs has been long defined, post-transcriptional events have recently emerged as critical regulators of the amplitude and specificity of the response. Regulation of mRNA stability [2], [3], translation [4], [5] or ubiquitination [6], [7] were shown to be critical for IFN-mediated antiviral effects. Nevertheless, the relative contribution of these post-transcriptional regulators and how they fine-tune the IFN system remain poorly understood.

Like other cytoplasmic RNA viruses, flaviviruses are highly sensitive to the antiviral effects of IFNs and as a result have evolved a wide array of countermeasures to avoid their action [8]. Described mechanisms include concealing double-stranded RNA replication intermediates in virally-induced ER membranes to decrease activation of innate immune sensors [9], cap methylation to mimic cellular mRNAs [10], degradation of regulators of IFN activation by the viral protease NS2B/3 [11], [12], or destabilization of transcription factor STAT2 by viral NS5 protein to dampen transcriptional activation of ISGs [13] More recently a ∼0.5 kb, abundant non-coding RNA derived from incomplete degradation of the viral 3′ untranslated region (3′UTR) by the cellular 5′-3′ exonuclease XRN1 and produced by all flaviviruses (termed sfRNA for subgenomic flaviviral RNA) was reported to be required for viral pathogenicity in a mouse model of the attenuated Kunjin strain (KUNV) of West Nile virus (WNV) [14]. Follow-up studies determined that KUNV sfRNA counteracted IFN antiviral activity [14], [15]. Strikingly, while the majority of genomes synthesized during infection are processed into sfRNA, it is dispensable for RNA replication in IFN-incompetent cells, arguing for an important, conserved role in antagonizing immune defenses. The only possible mechanism for the anti-IFN action of the sfRNA was suggested by a recent report that suggests the Japanese encephalitis virus (JEV) sfRNA inhibits IFN production by blocking the phosphorylation of IRF-3 [16]. The observation suggesting a decrease in IFN production by transfecting JEV sfRNA was not properly controlled by the use of other RNAs and thus we believe that to date the anti-IFN mechanism of sfRNA remains unknown.

As the flaviviral positive-strand 11 kb RNA genome encodes only 10 viral proteins, it is not surprising that host proteins, especially RNA binding proteins (RBPs), play a critical role in viral replication and pathogenicity. In a screen for host proteins interacting with dengue virus 2 (DENV-2) RNA, we identified ubiquitous, multifunctional RBPs, G3BP1, G3BP2 and CAPRIN1 [17]. G3BP1 and CAPRIN1 had been reported as proviral factors in vaccinia virus (VACV) and respiratory syncytial virus (RSV) infection [18], [19]. On the other hand, G3BP1 and G3BP2 had antiviral activity against poliovirus (PV) and alphaviruses [20], [21], suggesting a variety of possible mechanisms of action in viral infections. In this study, we investigated their role in DENV-2 infection. We found that these proteins have a potent antiviral action against flaviviruses, linked to a previously unknown role in regulating translation of ISG mRNAs. We further demonstrate that this activity is targeted by DENV-2 sfRNA, which binds G3BP1, G3BP2 and CAPRIN1 and prevents their function, protecting viral replication against IFN-mediated antiviral effects.

## Results

### G3BP1, G3BP2 and CAPRIN1 are novel mediators of the IFN response

G3BP1, G3BP2 and CAPRIN1 were initially discovered as interacting with DENV-2 3′UTR in an RNA affinity chromatography screen performed in our laboratory [17]. The variety of cellular functions described for these proteins in control of mRNA translation and stability, regulation of cell signaling pathways, and in the integrated stress response [22]–[26] prompted us to examine their role in DENV-2 infection.

RNAi-mediated knockdown and overexpression studies indicated that G3BP1, G3BP2 and CAPRIN1 had a modest but significant antiviral activity against DENV-2 NGC in HuH-7 cells (**Figure S1A–D**). Importantly, transfection of control and specific siRNAs did not induce IFN mRNA accumulation, which could have influence DENV-2 replication as an off target effect, [27] (**Figure S1E**). The antiviral effect was not restricted to the laboratory adapted DENV-2 NGC as G3BP1, G3BP2 and CAPRIN1 had antiviral activity against the clinical DENV-2 isolate PR1940 as well as the related yellow fever vaccine strain (YFV-17D) (**Figure S1F**). Nonetheless, G3BP1, G3BP2 and CAPRIN1 depletion had no effect on the DENV-2 strain PR6913, which exhibited low replication levels (**Figure S1F**). Since the type I IFN response has been long established as a critical mediator of anti-flaviviral innate immunity [8] and low levels of flaviviral replication have been shown to correlate with lower induction of type I IFN response [28] the data above suggested that G3BP1, G3BP2 and CAPRIN1 could play a previously unidentified role in the innate immune response.

**Figure 1:**
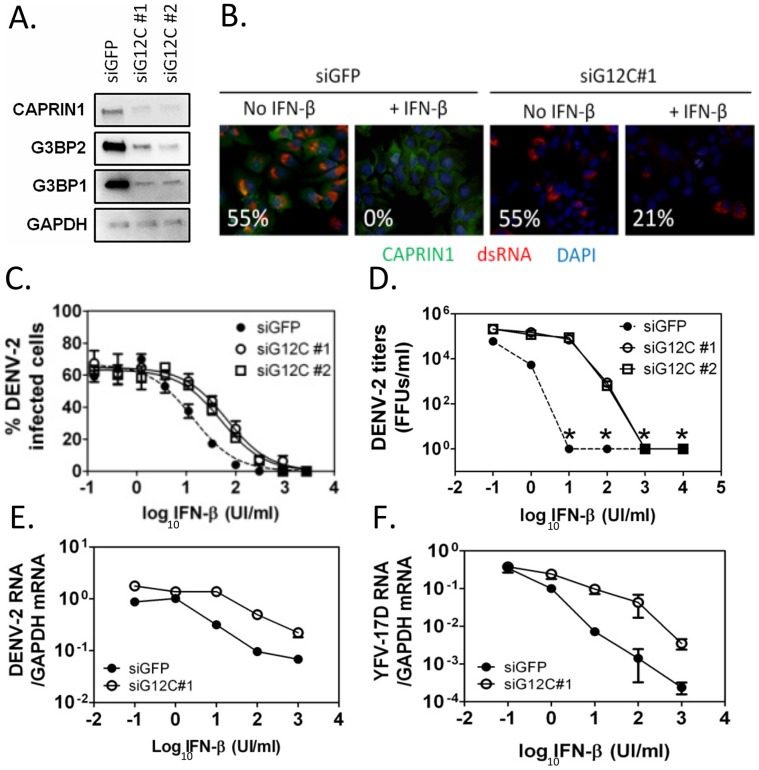
G3BP1, G3BP2 and CAPRIN1 are required for IFN-β mediated antiviral activity against DENV-2. (A) HuH-7 cells were treated with siGFP or one of two independent sets of siRNAs (siG12C#1 and siG12C#2) targeting G3BP1, G3BP2 and CAPRIN1, and knockdown was confirmed by western blot analysis. (B) Indirect immunofluorescence was used to detect dsRNA-containing replication complexes (red) and CAPRIN1 (green) in HuH-7 cells treated with control siGFP or siRNA targeting G3BP1, G3BP2 and CAPRIN1 (siG12C#1) and pretreated or not with 100 UI/ml IFN-β before infection with DENV-2 at MOI = 1 for 24 h. The percentage of cells with a replication complex (i.e., infected cells) for each condition is indicated in the lower left corner of each image. (C and D) Cells treated with siGFP, siG12C#1 or siG12C#2 were incubated with increasing concentrations of IFN-β for 16 h before DENV-2 infection at MOI = 1. DENV-2 infectivity was determined at 24 h post-infection by indirect immunofluorescence (C) and DENV-2 infectious particles production (D). Asterisks indicate values below detection levels. (E and F) HuH-7 cells treated with control or siG12C#1 siRNAs, pretreated with IFN-β as above and infected with DENV-2 (E) or YFV-17D (F) at MOI = 1. Viral RNAs levels were determined at 24 h post-infection by quantitative real-time RT-PCR and normalized to intracellular GAPDH mRNA levels.

In order to investigate a link between IFN-mediated antiviral activity and that of G3BP1, G3BP2 and CAPRIN1, cells depleted of these three proteins (**Figure 1A**) were pretreated with IFN-β before infection with DENV-2 NGC. As in previous studies [29], 100 UI/ml IFN-β, which is comparable to that observed in sera of DENV infected patients [30], abrogated formation of viral replication complexes (**Figure 1B**). Strikingly, the IFN effect was dramatically diminished in G3BP1, G3BP2 and CAPRIN1-depleted cells (**Figures 1B**), indicating that G3BP1, G3BP2 and CAPRIN1 are required for IFN-β antiviral effects.

Next, DENV-2 infectivity was measured over a range of IFN concentrations, revealing that treatment with two independent sets of siRNAs targeting G3BP1, G3BP2 and CAPRIN1 (siG12C#1 and siG12C#2) resulted in a 4- to 5-fold increase in IFN-β IC50 (**Figure 1C**). This effect was even more pronounced at the level of infectious progeny virus formation and accumulation of viral RNAs (**Figures 1D and 1E**), consistent with IFN-β targeting various steps of the viral life cycle. Notably, the magnitude of the G3BP1, G3BP2 and CAPRIN1-mediated antiviral activity in the presence of IFN-β was much greater than its ∼2-fold effect in the absence of IFN-β, (**Figure S1B**), suggesting that the main antiviral role of these proteins occurs through IFN action. It should be noted that HuH-7 cells secrete low levels of IFN-β upon DENV-2 infection ([31] and see below), and therefore conditions without exogenously added IFN-β should be consider to have low levels of IFN. Interestingly, G3BP1, G3BP2 and CAPRIN1 antiviral activity was redundant since depletion of all three proteins was required to observe an effect on DENV replication (**Figure S2**), which was consistent with previous studies on other functions of these RBPs [18], [21], [25], [32]. The requirement of G3BP1, G3BP2 and CAPRIN1 in IFN-mediated antiviral activity was observed in YFV-17D infection (**Figure 1F**). Taken together, these data indicate that G3BP1, G3BP2 and CAPRIN1 have an unexpected and important role in mediating the anti-flaviviral activity of IFNs.

### G3BP1, G3BP2 and CAPRIN1 are required for accumulation of ISG proteins

We next investigated the mechanism of action of this unexpected role of G3BP1, G3BP2 and CAPRIN1 in the IFN response. Since G3BP1, G3BP2 and CAPRIN1 had previously been determined to be critical regulators of stress granules (SG) assembly, another aspect of the cellular response to infection, we examined SG formation in infected cells and upon IFN treatment. Indeed, a recent study of SG dynamics in HCV infection revealed that treatment of HCV-infected cells with IFN-α triggered potent SG formation [33], suggesting that SG could mediate IFN antiviral effects. We monitored SG formation during DENV-2 infection in the presence or absence of IFN-β and found no evidence of increased SG formation in DENV-2 infected cells, even following treatment with IFN-β that effectively reduced viral replication (**Figure S3**). These data indicate that *bona fide* SG formation, which is defined microscopically by the appearance of cytoplasmic granules containing a set of protein markers, was not required to mediate the IFN anti-DENV-2 effects. Our data do not exclude an important role for SG and SG-associated proteins in DENV-2 infection, but they suggested that G3BP1, G3BP2 and CAPRIN1 could play an alternative role in the IFN response to DENV-2 infection.

**Figure 2:**
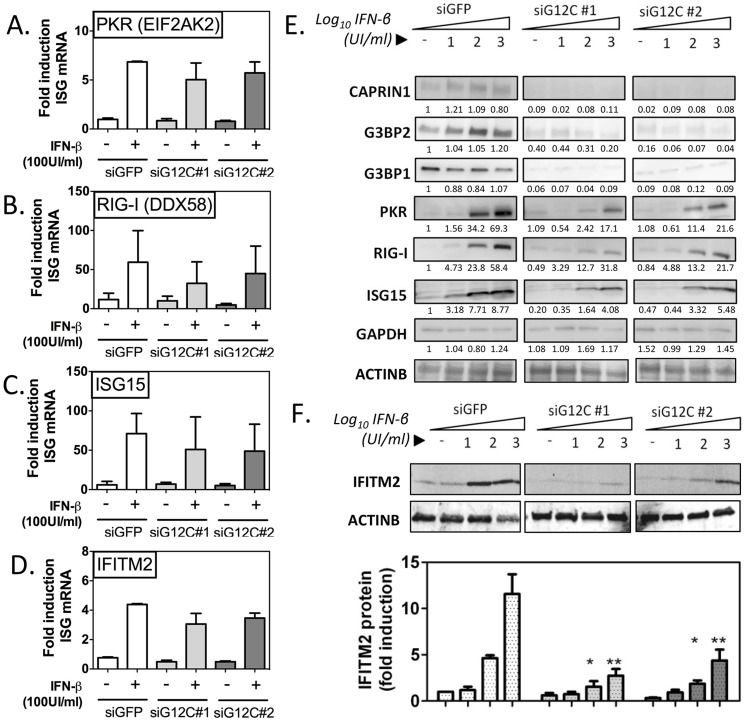
G3BP1, G3BP2 and CAPRIN1 regulate establishment of the antiviral state. HuH-7 cells treated with siGFP, siG12C#1 or siG12C#2 were stimulated with the indicated concentration of IFN-β for 16 h and ISG mRNA and protein levels were determined. (A–D) Cells were treated with 100 UI/ml IFN-β and PKR, RIG-I, ISG15 and IFITM2 mRNA levels were determined by quantitative real-time RT-PCR, normalized to intracellular GAPDH mRNA and expressed as fold induction compared to control untreated cells. (E) Cells were stimulated with 0, 10, 100 or 1000 UI/ml IFN-β and levels of proteins G3BP1, G3BP2, CAPRIN1 and representative ISGs, PKR (EIF2AK2), RIG-I (DDX58) and ISG15 were analyzed by western blots. Band intensity was determined by densitometry analysis using ImageJ and normalized to ACTINB. Results are presented as one representative experiment. (F) Cells were treated as in (E) and IFITM2 levels quantified in three independent experiments by measuring fluorescence intensity, using the Licor Odyssey system.

To gain insight into such alternative modes of action, we examined the integrity of the pathway leading to the establishment of the IFN induced antiviral state. Binding of IFNs to their receptor on the cell surface activates the JAK-STAT signaling cascade, leading to the transcriptional activation of hundreds of IFN-stimulated genes (ISGs), which have specific antiviral activities [34]. We selected a representative panel of ISGs and measured their induction in response to IFN-β in control versus G3BP1, G3BP2 and CAPRIN1 depleted HuH-7 cells. IFN-inducible IFITM2, RIG-I/DDX58 ISG15 and STAT1 have been reported to have anti-DENV-2 activity; however, the dsRNA-activated kinase PKR/EIF2AK2 and MX1 do not affect DENV-2 replication [35]–[38]. Quantitative real-time RT-PCR analysis showed no significant decrease of control mRNA levels (GAPDH or ACTINB) or ISG mRNA induction in response to increasing IFN-β concentration in G3BP1, G3BP2 and CAPRIN1-depleted cells (**Figures 2A to 2D** **and S4A to S4D**). Strikingly, expression of all six ISG proteins, however, was significantly reduced in G3BP1, G3BP2 and CAPRIN1-depleted cells (**Figures 2E, 2F** **and S4E**).

PKR protein accumulation, as measured by densitometry analysis of the western blot data shown in Figure 2E, was reduced 4.05 and 3.21 fold compared to siGFP control in siG12C#1 and siG12C#2-treated cells, respectively. In this experiment, RIG-I accumulation was reduced 1.86 and 1.80 fold, and ISG15 2.15 and 1.60 fold respectively. STAT1 protein levels were reduced 4.50 and 4.98 fold, and MX1 2.46 and 11.2-fold respectively in cells depleted of G3BP1, G3BP2 and CAPRIN1 (**Figure S4**). Because determination of protein levels from HRP-based western data is semi-quantitative, we performed analysis of IFITM2 levels using fluorophore-conjugated secondary antibodies in the Licor Odyssey system. Quantification of western blots by fluorescence intensity in three independent experiments revealed a 3- to 5-fold reduction in IFITM2 levels, normalized to ACTINB levels, after stimulation with 100 or 1000 UI/ml IFN-β (**Figure 2F**). These data indicate a general and robust effect of G3BP1, G3BP2 and CAPRIN1 on establishment of the antiviral state through post-transcriptional control of multiple ISGs. We propose that many more ISGs will be affected, and the cumulative effect would likely explain the dramatic drop in IFN antiviral potency observed in the previous experiments.

### G3BP1, G3BP2 and CAPRIN1 are critical regulators of ISG mRNA translation

**Figure 3:**
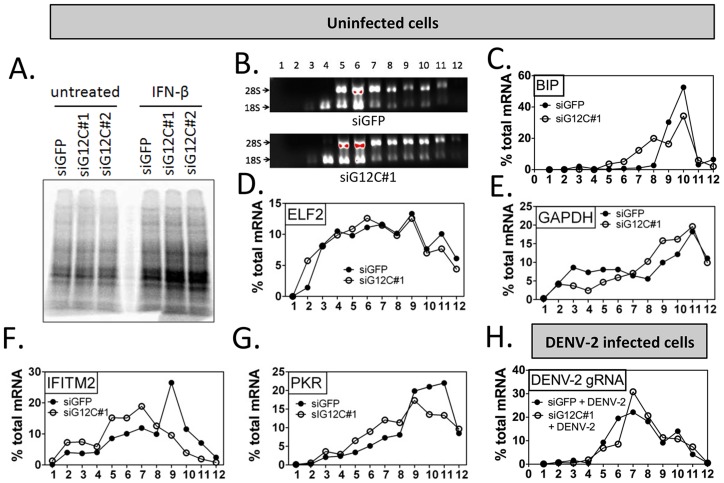
G3BP1, G3BP2 and CAPRIN1 are required for association of ISG mRNAs with polyribosomes. (A) HuH-7 cells treated with control siGFP or two independent sets of siRNAs against G3BP1, G3BP2 and CAPRIN1 and treated or not with 100 UI/ml IFN-β for 4 h were labeled with ^35^S metabolic for 1 h. Cell lysates were separated on a 4–15% SDS-PAGE gel and radioisotope incorporation assessed using a phosphorimager. (B–G) HuH-7 cells treated with control siGFP or siG12C#1 siRNAs were stimulated with 100 UI/ml of IFN-β for 4 h and cell lysates separated by velocity sedimentation on a 10–50% sucrose gradient. (B) Total RNA from each gradient fraction was separated on a 1% denaturing agarose gel and stained with EtBr. 28S and 18S ribosomal RNAs are indicated. (C–G) Levels of BIP, ELF2, GAPDH, IFITM2, and PKR mRNAs were determined by quantitative real-time RT-PCR and each fraction expressed as percentage of total for the specific mRNA in all fractions. Profiles result from one out of three representative experiments; for comparison, another replicate of these conditions can be found in Figure 4F–4I. (H) HuH-7 cells treated with siGFP or siG12C#1 and infected with DENV-2 (MOI = 1) for 24 h were stimulated with 100 UI/ml and processed for polyribosome fractionation as in (B). The percentage of DENV-2 genomic RNA in each fraction was measured by quantitative real-time RT-PCR.

Given our data above it was possible for G3BP1, G3BP2 and CAPRIN1 to influence ISG mRNA splicing, transport or translation, or ISG protein stability. Because of previous reports on these proteins [23], [24], [26], [39], [40], we first addressed their action on ISG mRNA translation. We focused on ISGs IFITM2 and PKR to delineate the role of G3BP1, G3BP2 and CAPRIN1. Using ^35^S metabolic labeling, we established that depletion of G3BP1, G3BP2 and CAPRIN1 depletion did not downregulate global cellular translation (**Figure 3A**). Consistent with a lack of a profound global effect on translation, polyribosome fractionation revealed no significant difference in rRNA profiles in G3BP1, G3BP2 and CAPRIN1-depleted cells (**Figure 3B**). Polyribosome association profiles of several cellular mRNAs (ELF2, GAPDH and BIP/GRP78 mRNAs) showed minimal or no change upon G3BP1, G3BP2 and CAPRIN1 depletion and IFN-β treatment (**Figures 3C–E**). Indeed, although GAPDH and BIP mRNA slightly shifted to lighter fractions, the fraction containing the majority of the mRNA remained unchanged, indicating that association of these mRNAs with polyribosomes was not dramatically affected. Polyribosome-association of IFITM2 and PKR mRNAs, however, was strongly impaired in the absence of G3BP1, G3BP2 and CAPRIN1 (**Figures 3F–G**), suggesting that the strong effect of G3BP1, G3BP2 and CAPRIN1 depletion was specific for ISG mRNA translation. Importantly, depletion of G3BP1, G3BP2 and CAPRIN1 did not affect polyribosome-association of DENV-2 genomic RNA (**Figure 3H**), supporting the previous hypothesis that G3BP1, G3BP2 and CAPRIN1 antiviral action is principally mediated by the IFN system rather than by a direct role in viral translation.

**Figure 4:**
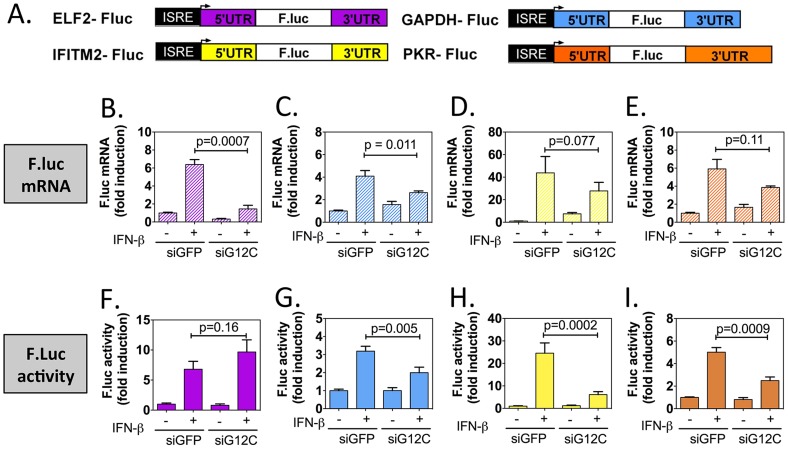
G3BP1, G3BP2 and CAPRIN1 depletion specifically inhibits translation of reporters under the control of ISG UTRs. (A) Schematic representation of IFN-stimulated response element (ISRE)-driven firefly luciferase reporters under the control of ELF2, GAPDH, IFITM2 or PKR UTRs. (B to I) HuH-7 cells stably transfected with the above constructs were treated with control siGFP or siG12C#1 siRNAs, induced with 1000 UI/ml of IFN-β for 10 h and firefly luciferase mRNA determined by quantitative real-time RT-PCR and normalized to GAPDH mRNA levels (B–E). Firefly luciferase protein levels were determined by measuring luciferase activity and normalized to total protein concentration (F–I). Both mRNA and protein activity are expressed as fold induction from control, untreated cells (siGFP, IFN-). Fluc measurements for the GAPDH-Fluc, IFITM2-Fluc and PKR-Fluc constructs were derived from 5 independent experiments in triplicate (n = 15). Fluc measurements for the ELF2-Fluc were derived from 3 independent experiments in triplicate (n = 9). All Fluc mRNA levels were measured in two of these independent experiments (n = 6).

In order to confirm the specificity of G3BP1, G3BP2 and CAPRIN1 in regulating ISG mRNA translation, we established stable cell lines expressing firefly luciferase reporters under the transcriptional control of a minimal promoter and an ISRE to provide IFN induction, and including the ELF2, GAPDH, IFITM2 or PKR UTRs with the first and last 30 nucleotides of the coding sequence (ELF2-Fluc, GAPDH-Fluc, IFITM2-Fluc and PKR-Fluc **Figure 4A**). We observed that IFN-induction of GAPDH-Fluc, IFITM2-Fluc and PKR-Fluc mRNAs was modestly reduced, although the effect was not significant for IFITM2-Fluc and PKR-Fluc. The induction of ELF2-Fluc mRNA was robustly and significantly reduced in the absence of G3BP1, G3BP2 and CAPRIN1 (**Figures 4B–E**). However, the significance of these observations is not clear since depletion of G3BP1, G3BP2 and CAPRIN1 did not affect absolute levels of endogenous GAPDH, IFITM2 or PKR mRNAs (see **Figures 2** **and** **3**). Importantly, IFN induction of ELF2-FLuc luciferase activity was not inhibited by knockdown of G3BP1, G3BP2 and CAPRIN1, however, IFN induction of IFITM2-Fluc and PKR-Fluc activity was robustly inhibited (**Figure 4F, H and I**). While induction of GAPDH-Fluc activity was significantly reduced in these conditions, the effect could be fully explained by the aforementioned effect on GAPDH-Fluc mRNA levels (compare **Figures 4C and G**). Indeed, a calculation of the relative translation efficiency, the ratio of protein induction (derived from luciferase activity) relative to mRNA induction, clearly revealed that both ELF2 reporter translation was increased 6.3-fold in G3BP1, G3BP2 and CAPRIN1-depleted cells compared to control siGFP, while the relative translation efficiency of IFITM2-Fluc and PKR-Fluc was reduced 2.54- and 1.3-fold, respectively (**Table 1**). In the case of GAPDH-Fluc, the relative translation efficiency was slightly reduced (1.07-fold), which correlates with the modest shift observed in GAPDH mRNA distribution in polyribosomes fractions in the absence of G3BP1, G3BP2 and CAPRIN1 (see **Figure 3E**).

**Table 1:** Summary of mRNA induction, protein induction and translation efficiency for each Fluc reporter construct.

Construct	Biological process	siGFP + IFN-β	siG12C#1 + IFN-β
ELF2-Fluc	mRNA induction	6.37±0.55	1.45±0.40***
	Fluc induction	6.80±1.02	9.67±1.56 (ns)
	Relative translation efficiency	1.06	6.66
			**+6.3**
GAPDH-Fluc	mRNA induction	4.04±0.49	2.63±0.15*
	Fluc induction	3.19±0.26	1.91±0.30**
	Relative translation efficiency	0.78	0.73
			**−1.07**
IFITM2-Fluc	mRNA induction	43.79±14.5	27.79±7.58 (ns)
	Fluc induction	24.55±4.53	6.12±1.32***
	Relative translation efficiency	0.56	0.22
			**−2.54**
PKR-Fluc	mRNA induction	5.91±1.06	3.85±0.17 (ns)
	Fluc induction	5.01±0.40	2.49±0.32***
	Relative translation efficiency	0.85	0.65
			**−1.3**

Results presented in Figure 4 were compiled and for each construct, the relative translation efficiency was calculated as the ratio of the average firefly luciferase activity induction (normalized to siGFP, untreated cells set as 1) over to the average firefly luciferase mRNA induction (normalized to siGFP, untreated cells set as 1). The fold difference between siGFP + IFN and siG12C +IFN is shown in bold in the fourth column. The p-values for the differences in mRNA and Fluc induction between siGFP + IFN-β and siG12C + IFN-β conditions are indicated: ns, non significant;

* p<0.05;

** p<0.01;

***p<0.005.

Taken together, these results show that G3BP1, G3BP2 and CAPRIN1 differentially affect reporter mRNA translation and that elements in the IFITM2 and PKR mRNA UTRs and/or the first and last 30 nucleotides of their coding sequence render translation of these messengers specifically dependent on G3BP1, G3BP2 and CAPRIN1. This suggest that G3BP1, G3BP2 and CAPRIN1 can, as previously described in the literature, play various roles in cellular mRNA metabolism, but are specifically required for translation of ISG mRNAs. While the precise mechanisms of translational regulation and how these proteins achieve selectivity remain to be investigated, several hypotheses will be proposed in the discussion.

### DENV-2 interferes with ISG mRNA translation

Flaviviruses, like other viruses, have been reported to interfere with the host IFN response by hijacking a large variety of cellular factors required for establishment of the antiviral state. In the case of DENV-2, all previously described evasion strategies affect signaling pathways upstream of ISG transcriptional activation [11], [13], [41], [42] However, these mechanisms are not completely efficient since ISG mRNA upregulation is observed widely in response to DENV-2 infection [43]. Data presented above suggested that ISG mRNA translation could be targeted by DENV-2 and this would not have been detected in previous studies measuring ISG mRNA induction as a surrogate for efficient IFN response.

**Figure 5:**
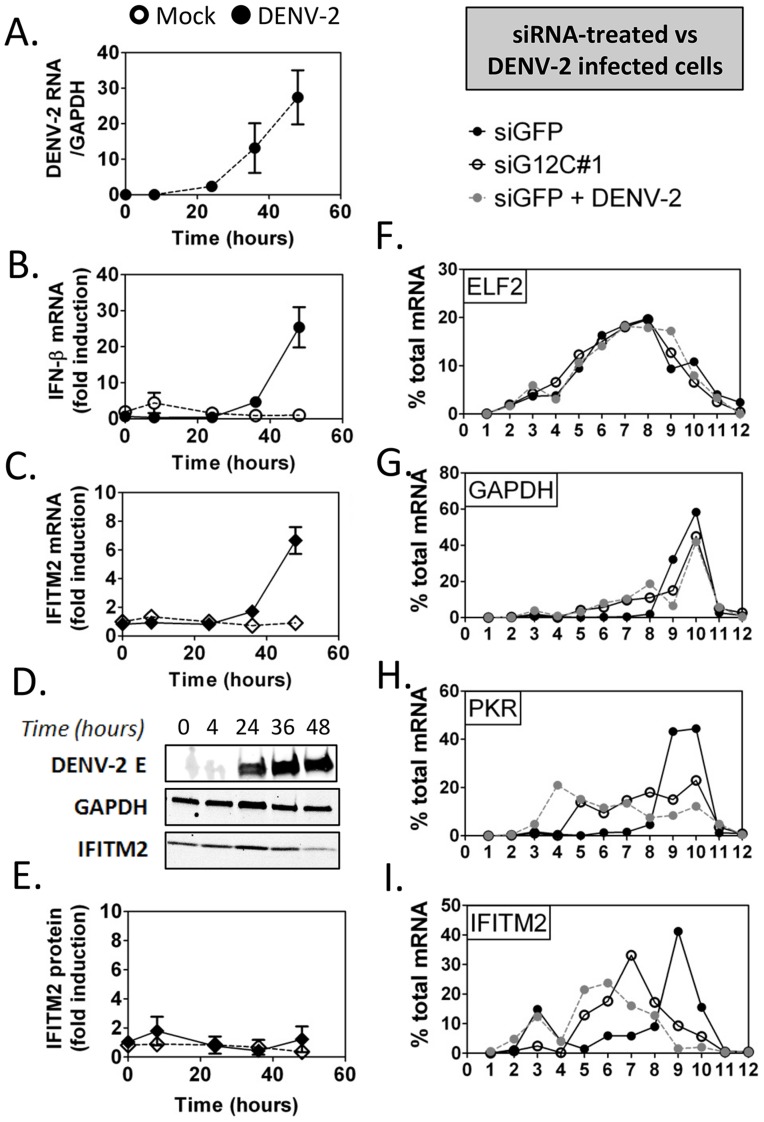
DENV-2 infection interferes with ISG protein expression. HuH-7 cells were infected with DENV-2 at MOI = 1 and levels of viral products and host IFN response mediators were determined at the indicated times post infection as described above. (A–C) DENV-2 genomic RNA and IFN-β and IFITM2 mRNAs were measured by quantitative real-time RT-PCR and normalized to GAPDH mRNA levels. (D–E) DENV-2 Envelope protein (E) and IFITM2 were detected by western blot and quantified by analysis of fluorescence intensity relative to GAPDH. Open symbols represent mock-infected cells and solid black or grey symbols DENV-2 infected cells for each time-course. (F–I) Control HuH-7 cells (siGFP, black), siG12C-treated (siG12C#1, open) or infected with DENV-2 at MOI = 1 for 24 h (siGFP + DENV-2, grey) and treated with 100 UI/ml IFN-β for 4 h were subjected to polyribosome fractionation. Percentage of ELF2, GAPDH, IFITM2 and PKR mRNAs across fractions was determined by quantitative real-time RT-PCR.

In DENV-2 infected cells, viral RNA replication resulted in a 24-fold increase in IFN-β mRNA between 24 and 48 h post infection, which was accompanied by a 7-fold increase in IFITM2 mRNA (**Figures 5A to 5C**). No induction of IFITM2 was detected up to 72 h post infection (**Figures 5D and 5E**), indicating that ISG expression was indeed controlled at a post-transcriptional level during DENV-2 infection. Importantly, polyribosome fractionation analysis showed that in DENV-2 infected cells and in G3BP1, G3BP2 and CAPRIN1-depleted cells, IFITM2 and PKR mRNA translation was strongly impaired (**Figures 5F to 5I**). Interestingly, DENV-2 infection inhibited IFN induction of both PKR mRNA and protein (**Figure S5**), indicating that this virus can regulate some ISGs via multiple mechanisms to keep the IFN response under check. Most importantly the data indicate that DENV-2 interfered with IFITM2 and PKR mRNA translation, which phenocopied G3BP1, G3BP2 and CAPRIN1 depletion, and suggested that DENV-2 gene product(s) target these RBPs.

### G3BP1, G3BP2 and CAPRIN1 interact with DENV-2 non-coding sfRNA during infection

Previously G3BP1 had been reported to be antagonized in poliovirus infection, where it is cleaved by a viral protease [20]. DENV-2 infection however, did not decrease the levels of G3BP1, G3BP2 or CAPRIN1 (**Figure S5**), suggesting a mechanism other than proteolytic degradation. We have shown previously that G3BP1, G3BP2 and CAPRIN1 each interact with the 3′UTR of DENV-2 RNA [17], a region included in the 3′UTR-derived non-coding sfRNA. These interactions, together with the fact that the sfRNA from a related flavivirus, Kunjin virus (KUNV), interferes with the IFN response [15], made DENV-2 sfRNA an ideal candidate for targeting G3BP1, G3BP2 and CAPRIN1. Therefore, we hypothesized that DENV-2 sfRNA would bind G3BP1, G3BP2 and CAPRIN1 and inactivate their antiviral effect.

**Figure 6:**
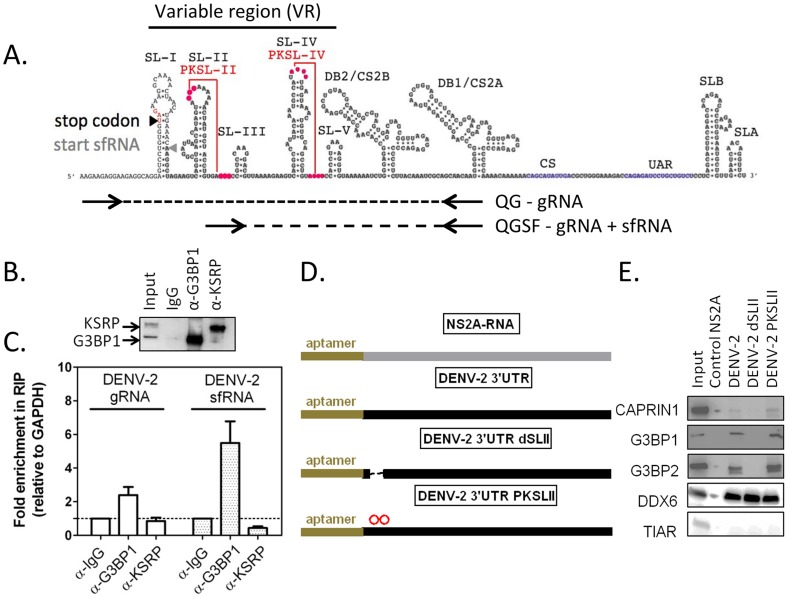
G3BP1, G3BP2 and CAPRIN1 interact with DENV-2 gRNA and sfRNA in infected cells. (A) The DENV-2 NGC 3′UTR variable region (VR) is predicted to contain five stem-loops (SL-I to SL-V) and two highly conserved pseudoknots PKSL-II and PKSL-IV. Sequences shared by DENV-2 gRNA and sfRNA are highlighted in grey. To relatively quantify these two RNAs species, a differential real-time RT-PCR strategy was designed in which one primer pair, QG, detects DENV-2 gRNA only, while the other primer pair, QGSF, amplifies sequences shared by the gRNA and sfRNA. sfRNA levels are obtained by subtraction of absolute levels of amplicons obtained from both primer pairs calculated against a standard curve (for more details refer to **Figure S7**). (B–C) HuH-7 cells were infected with DENV-2 at MOI = 1 for 24 h and binding of host RBPs to viral and cellular RNAs lysates was analyzed by RNA immunoprecipitation (IP). (B) A representative western blot shows robust enrichment of G3BP1 and KSRP in the specific IP. (C) Pellet fractions from IP with anti-G3BP1 or anti-KSRP antibodies were analyzed for DENV-2 gRNA and sfRNA as described above. Results are presented as mean ± SEM of the ratio of aforementioned RNAs over GAPDH mRNA in the pellet fraction, normalized to same value for control α-IgG IP. (D–E) RNAs containing a 5′ terminal binding aptamer were used to identify sequences required for G3BP1, G3BP2 and CAPRIN1 binding. RNA matrices DENV-2 3′UTR, the same deleted of SL-II (dSLII), or containing two point mutations in the terminal loop of SLII, which are predicted to disrupt PKSLII (D) were incubated with uninfected cell lysates and bound host RBPs eluted as previously described. The DENV-2 NS2A ORF was used as a negative control [17]. The binding of TIAR (TIAL1), DDX6, G3BP1, G3BP2, or CAPRIN1 was interrogated using specific antibodies. DDX6, which was shown to bind DENV-2 DB region, was used as a positive control [17]. TIAR, which was shown not to interact with DENV-2 positive strand RNA, was used as a negative control [64]. One representative western blot (E) of three done is shown.

In order to determine whether DENV-2 sfRNA interacts with G3BP1, G3BP2 and CAPRIN1, we first performed co-localization experiments using *in situ* hybridization for DENV-2 RNAs and immunofluorescence for G3BP1 in infected cells. *In situ* probes detecting the viral 5′UTR, which interrogate only the gRNA, and 3′UTR, which detect both gRNA and sfRNA, were both found to colocalize with G3BP1 during infection (**Figure S6**). To test and quantify an interaction between viral RNAs and the three RBPs, we used RNA-immunoprecipitation and a real-time PCR strategy designed to discriminate between gRNA and sfRNA, which is identical to the last 428 nucleotides of the genome [44] (**Figures 6A** **and S7A–E**). As suggested previously [44], we found that DENV-2 sfRNA was 5–10 times more abundant than the gRNA during infection of HuH-7 cells (**Figure S7G**). Both DENV-2 gRNA and sfRNA were found enriched in G3BP1-immunoprecipitates from infected cells (3- and 6-fold relative to GAPDH RNA, respectively), but were not found to interact with KSRP, an unrelated host RBP (**Figures 6B** **and** **5C**). DENV-2 gRNA and sfRNA were also enriched in G3BP2 and CAPRIN1 immunoprecipitates, while c-Myc mRNA was not enriched in these (**Figure S8**), confirming the specificity of the interaction. Taken together, these data indicate that G3BP1, G3BP2 and CAPRIN1 interact with DENV-2 gRNA and sfRNA during infection.

Having established that G3BP1, G3BP2 and CAPRIN1 interacted with DENV-2 sfRNA in infected cells, we sought to examine which sequence or structural elements were required for this interaction. The DENV-2 3′UTR contains a series of highly conserved secondary structures (**Figure 6A**), which have been proposed to serve as platforms of interaction for host RBPs [17] [45]. We designed sfRNA variants containing various deletions and point mutations (**Figure 6D**) and tested their ability to interact with G3BP1, G3BP2 and CAPRIN1 by RNA affinity chromatography. We found that stemloop II (SL-II), but not the predicted pseudoknot PKSL-II, was required for G3BP1, G3BP2 and CAPRIN1 binding to DENV-2 3′UTR (**Figure 6E**). We also tested the ability of the 3′UTR of related flaviviruses to interact with these RBPs and observed that only 3′UTRs of clinical isolates from DENV-2, but not DENV-3, the attenuated WNV subtype KUNV or the YFV vaccine strain 17D were able to pull-down G3BP1, G3BP2 and CAPRIN1 (**Figure S9**). Notably, in these mutants and isolates, the three proteins shared the same binding requirements, suggesting that these proteins interact as a complex.

### DENV-2 sfRNA binding to G3BP1, G3BP2 and CAPRIN1 downregulates ISG mRNA translation

**Figure 7:**
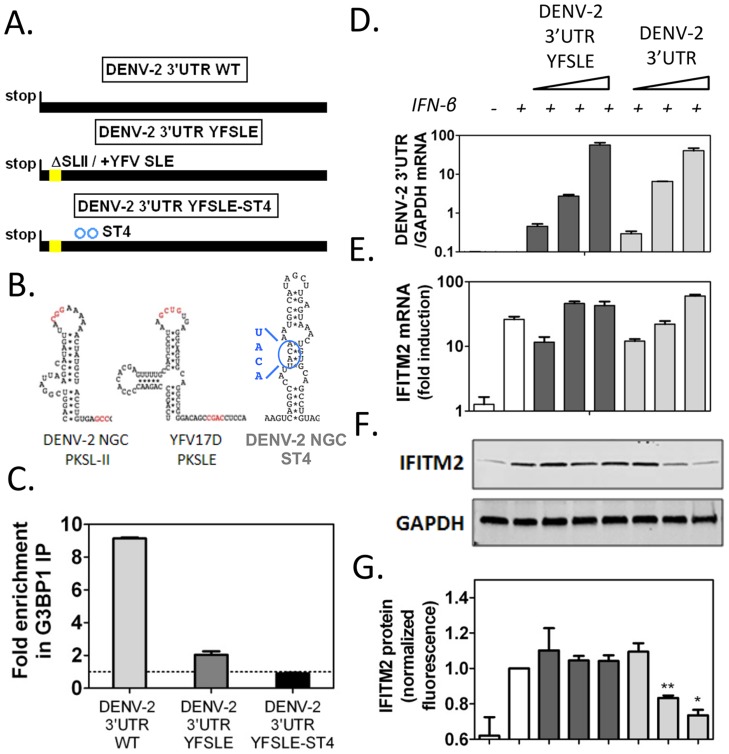
DENV-2 3′UTR downregulates ISG protein expression through G3BP1, G3BP2 and CAPRIN1 binding. (A) Schematics of DENV-2 3′UTR RNAs synthesized to mimic sfRNAs: DENV-2 3′UTR WT, DENV-2 3′UTR YFSLE replaced SL-II/PKSLII with the equivalent structure in YFV-17D (YFV-17D SLE, see panel B) in the DENV-2 3′UTR background, and DENV-2 3′UTR YFSLE-ST4 which contains an additional four point mutations in the middle stem of SL-IV. The corresponding structures are shown in (B). Nucleotides implicated in pseudoknot formation are highlighted in red and substitutions in DENV-2 SL-IV in blue. (C) *In vitro* transcribed DENV-2 3′UTR RNAs were transfected into HuH-7 cells and their binding to G3BP1 was interrogated by G3BP1 IP. Results are presented as mean ± SEM of three independent experiments, normalized to control IgG IP. (D–G) HuH-7 cells were transfected with 5 ng, 50 ng or 500 ng of *in vitro* transcribed DENV-2 3′UTR or DENV-2 3′UTR YFSLE, and treated with 100 UI/ml IFN-β for 4 h. (D–E) Levels of viral 3′UTR and IFITM2 RNAs were measured by quantitative real-time PCR and normalized to intracellular GAPDH mRNA levels. (F–G) Levels of IFITM2 protein were determined by western blots and quantified by analysis of fluorescence intensity relative to GAPDH. All results are presented as mean ± SEM of three independent experiments in triplicate. One representative western blot used for IFITM2 protein quantification is shown.

In order to determine whether DENV-2 sfRNA was able to inhibit G3BP1, G3BP2 and CAPRIN1 activity and impair ISG expression, we transfected increasing amounts of DENV-2 3′UTR RNAs, as sfRNA-mimics, into cells and measured ISG mRNA and protein expression upon IFN-β treatment. To control for effects mediated by functions of the sfRNA unrelated to G3BP1, G3BP2 and CAPRIN1 binding, we constructed a mutant unable to bind these RBPs but containing all other sequence elements of DENV-2 sfRNA. Since the structure required for binding, SL-II, has been implicated in formation and stability of flaviviral sfRNAs [46], we sought to minimize the effects of its deletion by replacing it with the equivalent structure from YFV-17D, SLE (**Figures 7A and 7B**), which did not interact with G3BP1, G3BP2 and CAPRIN1 *in vitro* (**Figure S9** and Ward et al, unpublished data). This hybrid mutant, DENV-2 3′UTR YFSLE, exhibited 5-fold decreased binding to G3BP1 (**Figure 7C**), and additional point mutations in DENV-2 SL-IV, whose secondary structure resembles SL-II (indicated on **Figure 7B**), further decreased the interaction to background levels (DENV-2 3′UTR YFSLE-ST4, **Figure 7C**).

When increasing amounts of *in vitro* transcribed RNAs were transfected into cells followed by treatment with 100 UI/ml IFN-β, we observed that DENV-2 3′UTR, but not the control DENV-2 3′UTR YFSLE RNA, was able to decrease in a dose-dependent manner expression of ISGs IFITM2 (**Figures 7D to 7G**) and PKR (**Figure S10**). Both RNAs accumulated to similar levels and had no effect on ISG mRNA induction levels (**Figures 7D, 7E** **and S10**), indicating that DENV-2 sfRNA is able to post-transcriptionally interfere with ISG expression and that this activity depended on G3BP1, G3BP2 and CAPRIN1 binding. As observed for G3BP1, G3BP2 and CAPRIN1 depletion, ectopic expression of DENV-2 3′UTR interfered with ISG mRNA association with polyribosomes, while GAPDH and ELF2 mRNA were minimally or not affected in the same conditions (**Figure S11**). Taken together, these data show that ectopic expression of DENV-2 sfRNA mimics inhibited IFITM2 and PKR mRNA translation through G3BP1, G3BP2 and CAPRIN1 binding.

### The G3BP1, G3BP2 and CAPRIN1-sfRNA interaction protects DENV-2 replicons from IFN-β

**Figure 8:**
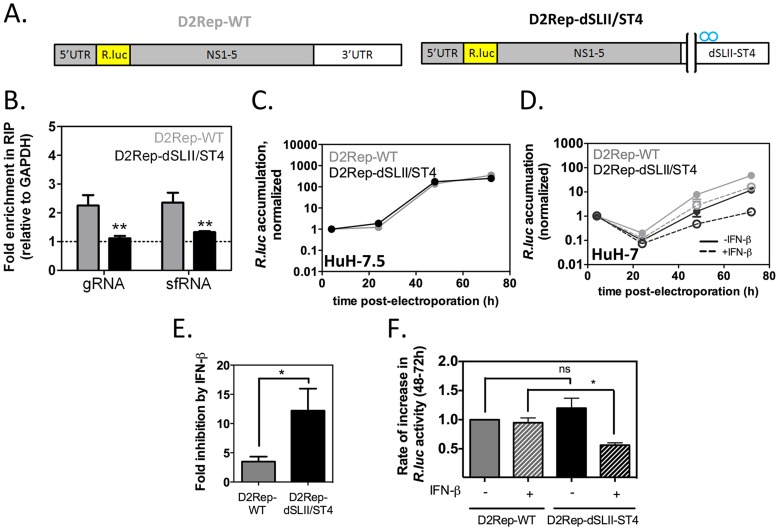
Binding to G3BP1, G3BP2 and CAPRIN1 protects DENV-2 replicons from the antiviral effects of IFN-β. *In vitro* transcribed DENV-2 reporter replicons (A) with wild-type 3′UTR or harboring a deletion of SL-II and the four point mutations in SL-IV as described in Figure 6A (D2Rep-WT, thereafter indicated with grey bars, and D2Rep-dSLII/ST4, black bars) were electroporated in HuH-7 cells. (B) Lysates were collected at 72 h post-electroporation and binding of viral RNA to G3BP1 determined by RNA-IP. (C) D2Rep-WT and D2Rep-dSLII/ST4 were electroporated in HuH-7.5 cells, lysates collected at different time-points and analyzed for *Renilla luciferase* reporter activity. Results are presented as mean ± sem of >2 independent experiments (D–F) HuH-7 cells electroporated with D2Rep-WT or D2Rep-dSLII/ST4 were treated or not with 50 UI/ml IFN-β at 4 h post-electroporation and *Renilla luciferase* reporter activity measured over time (D). Based on (D), the inhibition of *Renilla luciferase* accumulation at the 72 h time-point in the presence vs in the absence of exogenously added IFN (E) was calculated for each construct and the rate of increase in *Renilla luciferase* activity between 48 and 72 h post-electroporation (F, normalized to that of D2Rep-WT in the absence of exogenously added IFN) was calculated for each condition. All results in HuH-7 are derived from three independent experiments, each comprising two to three independent electroporations per reporter.

We showed that interaction of DENV-2 sfRNA with G3BP1, G3BP2 and CAPRIN1 was able to downregulate expression of ISGs, which is consistent with the sfRNA acting as a decoy for these host RBPs. To further test this hypothesis and determine the importance of this mechanism during infection and for viral evasion of the IFN response, we constructed mutant DENV-2 replicons unable to sequester G3BP1, G3BP2 and CAPRIN1. We used the established DENV-2 replicon system [47], whose biphasic reporter activity examines translation of input RNAs and subsequent replication and translation steps independently, to evaluate the effect of G3BP1, G3BP2 and CAPRIN1 binding on translation, replication and sensitivity to inhibition by IFN-β. We modified the DENV-2 replicon 3′UTR deleting the SL-II and introducing point mutations in SL-IV described before (D2Rep-dSLII-ST4, **Figure 8A** **, S12A**) and confirmed that replicon RNAs bearing these mutations had reduced binding to G3BP1 (**Figure 8B**). While SLII was reported to be required for sfRNA formation in some flaviviruses, we did not measure a decrease in sfRNA formation in dSLII-ST4 mutants (**Figure S12B**), ruling out the possibility that the effect of the mutation could be linked to SL-II functions mediated by other regions of the sfRNA. Indeed this finding is consistent with *in vivo* results in the recent report by Liu et al [48]. We electroporated these reporters into HuH-7.5 cells, which were derived from HuH-7 cells and harbor a point mutation in RIG-I that renders them deficient in IFN production through this pathway [49] and the parental HuH-7 cells. Importantly, we detected no difference in luciferase activity between D2Rep-WT and D2Rep-dSLII/ST4 at any time after electroporation (**Figure 8C**), indicating that reduced binding to G3BP1, G3BP2 and CAPRIN1 had no effect on replicon translation or replication. In HuH-7 cells the dSLII-ST4 mutation did not alter very early luciferase activity, a measure of translation of input RNAs (**Figure S12C**), however luciferase activity of the D2Rep-dSLII/ST4 was reduced by an average of 4.4-fold compared to the WT replicon at 72 h post-electroporation (**Figure 8D**). The different effects in HuH-7.5 and HuH-7 cells suggested that G3BP1, G3BP2 and CAPRIN1 binding to viral RNAs was required for viral replication in the context of a functional innate immune response.

To examine the effect of adding exogenous IFN on D2Rep-WT and D2Rep-dSLII-ST4 activity we electroporated these in HuH-7 cells and treated these with 50 UI/ml IFN-β at 4 h post-electroporation. The modest deleterious effect of the dSLII/ST4 mutation was strikingly enhanced by IFN treatment, with luciferase activity reduced 16.9-fold compared to the D2Rep-WT at 72 hr post-electroporation (**Figure 8E**). Overall, addition of exogenous IFN-β inhibited D2Rep-WT activity by 3.2-fold at 72 h post-electroporation while D2Rep-dSLII-ST4 was inhibited 12.3-fold (**Figure 8F**), indicating that the dSLII/ST4 mutation renders replicons more sensitive to the antiviral effects of IFNs. Finally, we analyzed the rates of accumulation of luciferase reporter between 48 and 72 h post electroporation. We observed no significant difference between the rates of D2Rep-WT and D2Rep-dSLII/ST4 in the absence of exogenously added IFN-β; however the D2Rep-dSLII/ST4 was significantly impaired in the presence of exogenously added IFN (**Figure 8G**). On the one hand, in the presence of low levels of endogenous IFN, which we expect with HuH-7 but not HuH-7.5 cells, after an initial delay the mutant replicon is still able to surmount IFN-mediated inhibition. On the other hand in the presence of higher levels of IFN the D2Rep-dSLII-ST4 is persistently inhibited. The results above convincingly argue that anti-DENV-2 action of G3BP1, G3BP2 and CAPRIN1 is mediated by their pro-IFN activity and support the hypothesis that the DENV-2 sfRNA antagonizes the IFN response in part by sequestering these host RBPs.

## Discussion

**Figure 9:**
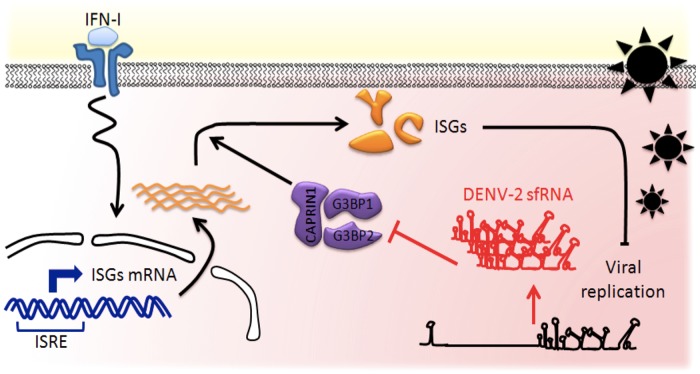
Model of the DENV-2 sfRNA antagonizing IFN action. IFNs are produced by infected cells and released in the extracellular space. Binding of IFN to IFNAR on neighboring naïve cells activates a cascade of signaling events leading to selective transcriptional activation of ISGs, which contain an interferon-sensitive response element (ISRE). Host RBPs G3BP1, G3BP2 and CAPRIN1 are required for translation of antiviral ISGs proteins and as a consequence for establishment of the antiviral state. In infected cells, high levels of non-coding sfRNA are produced and act as a RNA sponge, binding to host RNA-binding proteins. G3BP1, G3BP2 and CAPRIN1 bound to DENV-2 sfRNA are prevented to exert their activity in post-transcriptional regulation, leading to downregulation of ISGs, thus protecting DENV-2 replication against IFN antiviral effects.

In this study we make two new and important observations in the understanding of host innate antiviral measures and their inhibition by viral countermeasures. First, we identified G3BP1, G3BP2 and CAPRIN1, three conserved, multifunctional RNA-binding proteins, as critical positive regulators of the antiviral IFN response. This unexpected role was mediated through the specific activation of antiviral ISG mRNA translation. Second, we described the DENV-2 sfRNA as an antagonist to their antiviral effect, providing the first mechanism of action for this abundant, non-coding flaviviral RNA (**Figure 9**).

Although G3BP1, G3BP2 and CAPRIN1 have been shown to have a large variety of cellular functions, this report associates them for the first time with innate immunity. We show that these three RBPs were required for an antiviral IFN response against several isolates of DENV-2 and YFV-17D. Our data indicate that G3BP1, G3BP2 and CAPRIN1 regulate the expression of ISGs known to have broad antiviral activity: PKR, RIG-I, IFITM2, ISG15, STAT1 and MX1 [34]. Therefore, while the full spectrum of ISG targets and the individual contributions of the three RBPs remain to be determined, we posit their antiviral activity will be conserved against a wide array of viruses. Indeed previous evidence suggested this: the poliovirus (PV) protease degrades G3BP1 [20]; the core protein of Japanese encephalitis virus (JEV), another flavivirus, was identified as an important CAPRIN1 antagonist [50], and the nsP3 protein of Chikungunya virus (CHIKV), an alphavirus, as G3BP1 and G3BP2 opponent [51]. All these interactions were shown to be required for optimal viral replication, supporting our conclusion that G3BP1, G3BP2 and CAPRIN1 are major regulators of the cellular immune response.

The fact that these RBPs were not previously identified as IFN-related antiviral factors can be explained by two reasons. First, previous studies usually focused on SG formation and were performed in absence of exogenously added IFN. Second, the role of these proteins was examined independently, in ways that would not unearth their redundant functions in the IFN system. Interestingly, the direct antiviral role of SG formation is intuitive but has not been formally demonstrated given the challenges in differentiating the role of the granules themselves from the role of their numerous individual components. While the relative contributions and connections of these two branches of the innate immune response remain to be determined, our study suggests that activity of G3BP1, G3BP2 and CAPRIN1 against DENV-2 is primarily through the IFN system.

Perhaps our most unexpected finding was that G3BP1, G3BP2 and CAPRIN1 are critical for ISG mRNAs translation, a step previously understudied in the IFN response. Although the dogma is that establishment of the IFN-mediated antiviral state is primarily controlled by transcriptional activation, recent evidence suggests that additional layers of control regulate the amplitude and specificity of the response. For instance, a screen for host proteins implicated in IFN-mediated inhibition of hepatitis-C virus identified a large number of splicing factors [52]. This result implicates post-transcriptional mechanisms, which could regulate splicing or the proteins could moonlight in other aspects of RNA metabolism. Control of the stability of IFN-β mRNA by KSRP and STAT mRNA by PCBP2 were equally able to modulate IFN-mediated inhibition of viral replication [2], [3]. A recent study implicates, but does not directly address, the importance of ISG translational regulation [53] in antiviral signaling and underscores the importance of our findings.

The precise mode of action of G3BP1, G3BP2 and CAPRIN1 in ISG translational regulation and especially how specificity for ISG mRNAs is achieved remain to be elucidated. Several hypotheses could be considered. G3BP1, G3BP2 and CAPRIN1 could bind to ISG mRNA UTRs and recruit translation initiation factors, recruit ISG mRNAs to subcellular localizations where translation is more efficient in conditions of stress, or relieve miRNA-mediated inhibition of ISG mRNA translation. The RBPs could also act indirectly either activating or repressing mRNAs coding for positive or negative regulators of ISG mRNA translation. Alternatively, G3BP1, G3BP2 and CAPRIN1 could modulate signaling events leading to translational activation in the IFN response, such as the PI3K/Akt or Mnk pathways that are required for ISG mRNA translation [5], [54]. Finally, the RBPs could be involved in a stress response induced by IFNs that while not inducing bona fide SG would generally repress many mRNAs and by mass action enhance the translation of ISG mRNAs. In all above scenarios though, the cis-acting elements in the ISG mRNA UTRs conferring dependency on G3BP1, G3BP2 and CAPRIN1 will be a critical feature to determine.

In the second part of our study, we show that the DENV-2 abundant, non-coding sfRNA interacts with G3BP1, G3BP2 and CAPRIN1, inactivates them and thus mediates inhibition of ISG expression. The sfRNA - G3BP1, G3BP2 and CAPRIN1 interaction that we propose as a decoy mechanism was conserved for DENV-2 clinical isolates indicating its potential relevance for DENV-2 pathogenicity. While the antagonism of the immune response by viral non-coding RNAs has been well described, few mechanisms of action have been uncovered. Sequestration of host proteins has been widely hypothesized but only in a few instances was it formally demonstrated. The adenovirus VA RNAs was shown to bind and antagonize PKR and a similar role was proposed for Epstein Barr virus EBER RNAs [55]–[57]; the Sendai virus trailer RNA was hypothesized to sequester the RBP TIAR to subvert apoptosis [58]; the interaction between Kaposi's sarcoma-associated herpesvirus (KSHV) PAN RNA and PABP was suggested to participate in the host translational shutoff effect [59], [60]. Here we demonstrate a role for DENV-2 sfRNA as a molecular sponge or decoy for G3BP1, G3BP2 and CAPRIN1 resulting in a crippled IFN response.

While the sfRNA-G3BP1, G3BP2 and CAPRIN1 interaction was conserved for all DENV-2 viruses tested, no binding was detected for DENV-3, KUNV or YFV-17D 3′UTR. This suggests that although the IFN antagonist action of the sfRNA is conserved among flaviviruses, the precise mechanisms diverge for different viruses [15]. This is not unexpected since specific tactics for viral evasion of the IFN response have been shown to vary widely between related viruses, even strains of the same virus [8], [34]. For instance, NS4B proteins from some DENV-2 clinical isolates, but not from others, were able to interfere with IFN signaling [61]. Furthermore the sfRNA includes the so-called variable region (VR), which, while generally conserved in RNA secondary and tertiary structure, diverges significantly in primary sequence among flaviviruses. The VR is therefore a propitious platform for rapid evolution of new host RBP binding sites providing this viral genus with a wide array of tactical solutions to counter host innate defenses. It is thus conceivable that KUNV sfRNA, although not binding to G3BP1, G3BP2 and CAPRIN1, could target different subsets of host RBPs to prevent establishment of the antiviral state.

To conclude, it is widely accepted that host immune measures and pathogen countermeasures evolve rapidly, leading to remarkable diversity on both sides. Here, we propose that RBPs such as G3BP1, G3BP2 and CAPRIN1 are critical mediators of the antiviral state and that antagonizing them is a strategy employed by many viruses, including DENV-2. Equally, we believe that targeting different subsets of host RBPs is a pan-flaviviral anti-IFN strategy, for which many targets remain to be uncovered.

## Methods

### Cells, viruses and infections

HuH-7 hepatocellular carcinoma cells were maintained in DMEM supplemented with 10% FBS. BHK-21 cells, which were used for virus titration, were maintained in RPMI supplemented with 10% FBS. DENV-2 strain NGC and YFV-17D were propagated in Aedes albopictus C6-36 cells. All infections were carried at a multiplicity of infection of 1 (MOI = 1) for 24 h unless otherwise indicated. Infectivity was measured using indirect immunofluorescence detection of viral antigens or dsRNA in infected cells, quantitative real-time RT-PCR analysis of viral genomes, or quantification of infectious particles released by focus forming assay, as previously reported [17].

### siRNA-mediated knockdown of gene expression

25 nM of the indicated siRNA duplexes or 75 nM control siRNA (see supplementary materials) were transfected into cells at 50% confluency twice at 48 hr intervals (day 1 and 3) with Lipofectamine RNAiMax (Invitrogen) following manufacturer's instructions. Human IFN-β (PBL Interferon Source) was added to cells 24 hrs post-transfection and incubated for 16 h prior harvesting or infection with DENV-2. Lysates were collected 24 hrs post infection (day 6) and analyzed by western blotting for knockdown efficiency and ISG expression, and quantitative real-time RT-PCR for RNA levels (see supplementary methods).

### Polyribosome fractionation

Polyribosome fractionation was performed as previously described [62] with minor modifications: cells were harvested by trypsinization and 50 µg/ml cycloheximide was added into polyribosome lysis buffer. Individual mRNA levels in each fraction were measured by quantitative real-time RT-PCR and expressed as percentage of total for this mRNA in all the gradient fractions.

### ISRE-luciferase reporters

pcDNA3.1 constructs containing the firefly luciferase open reading frame flanked by IFITM2, PKR, GAPDH, or ELF2 5′ and 3′UTRs and driven by an ISRE promoter (for cloning details refer to supplementary materials) were transfected in HuH-7 cells and selected for stable expression in DMEM supplemented with 1500 µg/ml G418 (Gibco). siRNA-mediated knockdown was performed as described and cells stimulated with 1000 UI/ml IFN-β for 10 h. Firefly luciferase activity was assessed using the Dual luciferase reporter assay system (Promega). Firefly luciferase mRNA levels were measured by quantitative real-time RT-PCR.

### Analysis of RNA-protein interactions

RNA-immunoprecipitations were performed using the MAGNA-RIP kit (Millipore) following manufacturer's recommendations. The levels of RNA in IP were determined by quantitative real-time RT-PCR and normalized to GAPDH mRNA levels and control rabbit IgG IP following the formula:
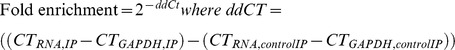
Tobramycin RNA affinity chromatography was carried out as described previously [17].

### Relative quantification of sfRNA levels

DENV-2 gRNA and sfRNA levels were quantified using a differential quantitative real-time RT-PCR assay designed based on the sfRNA mapping in Liu et al [44] (see Figure 5 and S7). One primer, annealing upstream of the stop codon in which one pair of primer recognizes specifically gRNA while a second pair of primers amplifies sequences shared between gRNA and sfRNA. Briefly, RNA extracted from experimental samples was reverse transcribed and parallel reactions set-up. Primer QG-FOR (5′ CCATGAAAAGATTCAGAAG 3′, annealing upstream of the stop codon) was used to detect gRNA only while primer QGSF-FOR (5′ GTG AGC CCC GTC CAA GG 3′, annealing downstream of the start of sfRNA) detected both gRNA and sfRNA. The reverse primer QGSF-REV (5′ GCTGCGATTTGTAAGGG 3′ annealing downstream of DB2) was shared, leading to products of 309 and 184 bp, respectively. In order to determine the relative sfRNA/gRNA ratio in a given sample, 1–2 µg of total cellular RNA (1–10 ng of in-vitro transcribed RNA) were incubated at 70°C for 5 min and reverse transcribed using the ImPromII kit (Promega) following manufacturer's recommendation. Triplicate wells containing 100–200 ng of cDNA, 300 pmol of each primer (QG-For or QGSF-For and QGSF-Rev) and Biorad SYBR Green reagent following manufacturer's recommendation were set up in a total of 25 µl. Reactions were run on a Biorad CFX96 quantitative real-time RT-PCR with the following parameters: 90°C 5 min, 40 repeats of 90°C for 30 s, 55°C for 30 s and 72°C for 30 s. Fluorescence detection was performed during the 72°C elongation step at each cycle. For each reaction the molar amount of template (n(G) and n(GSF)) was calculated from the CT value using a standard curve generated from serial dilutions of reverse transcribed purified full-length D2Rep RNA. sfRNA levels were inferred by subtracting the molar amount n(GSF) – n(G). A similar strategy was designed for analysis of YFV-17D gRNA and sfRNA levels, in this case primers were based on sfRNA mapping in Silva et al [63]. (YFV-G-For 5′ GGATGGAGAACCGGACTCC 3′, YFV-GSF-For 5′ GCTAAGCTGTGAGGCAGTGC 3′, YFV-GSF-Rev 5′ CGTCTTTCTACCACCACGTG 3′).

### DENV-2 3′UTR transfections

Templates for synthesis of control DENV-2 3′UTR YFSLE, in which the DENV-2 SL-II sequence (DENV-2 nt 10306–10348) was replaced by YFV 17D SLE sequence (YFV17D nt 10530–10611), were custom synthesized by GenScript. DENV-2 3′UTR and 3′UTR YFSLE templates were PCR amplified from stock plasmids to add a T7 promoter immediately upstream of the DENV-2 stop codon (T7-VR-For), and *in vitro* transcribed using the MegaScript kit (Ambion). 10, 100 or 1000 ng/ml RNA were transfected in cells at 50% confluency using Lipofectamine RNAiMax for 4 h. Cells were washed and incubated with complete medium containing 100 UI/ml IFN-β for 4 h before analysis of protein and mRNA contents.

### DENV-2 replicon

The DENV-2 reporter replicon system (D2Rep), based on DENV-2 strain 16681 (U87411.1), has been described before [47]. Detailed experimental procedures are available in supplementary materials.

### Statistical analysis

All results are presented as mean ± SEM of at least 3 independent experiments, unless otherwise indicated. Data were analyzed using unpaired, two-tailed Student's t-test and considered significant if p<0.05 (*p<0.05; **p<0.01; ***p<0.005).

### Accession numbers

The following reference sequences were used to design oligonucleotides throughout the study: DENV-2 NGC (AF038463.1); DENV-2 PR1940 (GQ398308.1); DENV-2 PR5344 (GQ398283.1); DENV-2 EDEN 05K3295 (EU081177.1); DENV-3 EDEN 05K802 (EU81184.1); DENV-3 EDEN 05K4454 (EU081222.1); YFV-17D (X03700.1); G3BP1 (NM_005754.2); G3BP2 (NM_203505.2); CAPRIN1 (NM_005898.4); IFITM2 (NM_006435.2); ISG15 (NM_005101.3); MX1 (NM_01144925.2); DDX58/RIG-I (NM_014314.3); EIF2AK2/PKR (NM_002759.3); STAT1 (NM_007315.3); GAPDH (NM_002046.3); ELF2 (NM_201999.2); GRP78/BIP (NM_005347.4).

## Supporting Information

Figure S1 **G3BP1, G3BP2 and CAPRIN1 have antiviral activity against DENV-2.** (A–B) HuH-7 cells were left untreated (NT) or treated with control siRNA (siGFP) or siRNA targeting G3BP1, G3BP2 and CAPRIN1 (siG12C#1) and infected with DENV-2 NGC at MOI = 1 on day 5. Knockdown efficiency and viral protein expression were determined by western blot for G3BP1, G3BP2, CAPRIN1 and DENV-2 envelope (E) protein (A); infectious particle production was measured by focus forming assay at 24 h post infection and expressed as percent of the untreated, infected control from three independent experiments (B). (C–D) Effect of overexpression of G3BP1 on DENV-2 infection. HuH-7 cells were transfected with plasmids expressing either control (GFP) or GFP-tagged G3BP1 and infected with DENV-2 NGC at MOI = 1. Recombinant protein expression and viral protein expression were determined by western blot for DENV-2 E and GFP (C). Infectious particle production was measured by focus forming assay at 24 h post infection and expressed as percent untreated, infected control from 3 independent experiments (D). Asterisks indicate values below detection levels. (E) HuH-7 cells were left untreated (NT) or treated with control siRNA (siGFP) or siRNA targeting G3BP1, G3BP2 and CAPRIN1 (siG12C#1) twice over the course of four days. Lysates were collected on day 5 and levels of IFN-β mRNA measured by quantitative real-time RT-PCR and normalized to intracellular GAPDH mRNA levels. (F) G3BP1, G3BP2 and CAPRIN1 antiviral activity against a panel of flaviviruses. HuH-7 cells were treated with the indicated siRNAs (siGFP or siG12C) and infected with DENV-2 NGC, clinical isolates DENV-2 PR6913 and PR1940 or YFV-17D at MOI = 1. Viral RNA levels were measured at 24 h post-infection by quantitative real-time RT-PCR, using DENV-2 gRNA primer pair for DENV-2 NGC, DENV-2 PR6913 and DENV-2 PR1940 (100% primer sequence identity) and YFV-17D gRNA primer pair for YFV-17D. Results were expressed as fold induction compared to uninfected cells and normalized to intracellular GAPDH mRNA levels. All results are presented as mean ± SEM from 3 independent experiments and analyzed using an unpaired two-tailed Student's t-test. *p<0.05 **p<0.01.
(PDF)

Figure S2 **Individual depletion of G3BP1, G3BP2 or CAPRIN1 does not impair IFN-β mediated antiviral activity.** HuH-7 cells were treated with control siRNA (siGFP, black), individual siRNAs targeting G3BP1, G3BP2 or CAPRIN1 (siG3BP1#1, siG3BP2#1, siCAPRIN1#1, purple, blue and green, respectively), or a pool of all three siRNAs (siG12C#1, red), pretreated with increasing concentrations of IFN-β and infected with DENV-2 at MOI = 1. Viral RNA levels were determined at 24 h post-infection by quantitative real-time RT-PCR and normalized to intracellular GAPDH mRNA levels. Results are presented as mean ± SEM of two independent experiments in duplicate.
(PDF)

Figure S3 **IFN-β mediated antiviral activity against DENV-2 is not linked to stress granule (SG) formation.** (A–C) DENV-2 inhibition by IFN-β is not accompanied by SG formation. Control or IFN-β treated HuH-7 cells were infected with DENV-2 at MOI = 1. The following IFN-β treatments were used: (#1) No IFN, (#2) 10 UI/ml 16 h prior to infection, (#3) 100 UI/ml 16 h prior to infection, (#4) 100 UI/ml 4 h after infection. Cells were fixed at 24 h post-infection and probed by indirect immunofluorescence for SG marker TIAL1 (TIAR, yellow) and DAPI (blue) (A). The percentage of SG-containing cells, defined as presenting more than 3 TIAR-containing cytoplasmic foci was determined manually (B – examples of positive cells are denoted by red arrows in panel A). The percentage of infected cells was determined by indirect immunofluorescence for dsRNA-containing replication complexes (C). Quantifications from one representative experiment (n>200 cells from one field) are shown. As described previously (ref), DENV-2 infection in control cells led to a slight induction of SG formation; pretreatment with increasing concentrations of IFN-β inhibiting DENV-2 replication did not affect the proportion of SG-containing cells nor did IFN-β treatment at 4 h post-infection, which did not affect viral replication. (D) The IFN-β response does not correlate with SG assembly. Cells pretreated with 100 UI/ml IFN-β and treated with SG inducers (50 mM sodium arsenite added to the media or 500 ng/ml polyI:C transfected with Lipofectamine 2000), or infected with DENV-2 were stained by indirect immunofluorescence for SG markers CAPRIN1 (green) and TIAR (red). The percentage of SG-containing cells (defined as cells with >3 CAPRIN1 and TIAR-containing cytoplasmic foci), was determined for >100 cells from one field and is indicated for each condition.
(PDF)

Figure S4 **G3BP1, G3BP2 and CAPRIN1 are dispensable for ISG mRNA induction but required for accumulation of MX1 and STAT1 proteins.** HuH-7 cells treated with siGFP, siG12C#1 or siG12C#2 were stimulated with the indicated concentration of IFN-β for 16 h and ISG mRNA and protein levels were determined. (A–B) IFITM2 and PKR mRNA induction upon treatment with 0, 10, 100 or 1000 UI/ml IFN-β was determined by quantitative real-time RT-PCR, normalized to intracellular levels of GAPDH mRNA and expressed as fold induction compared to control, untreated cells. (C–D) The same method was applied to MX1 and STAT1 mRNA induction upon treatment with 100 UI/ml IFN-β. (E) Protein levels of MX1, STAT1 and ACTINB were analyzed by western blot using HRP-conjugated secondary antibodies. Band intensity was determined by densitometry analysis using ImageJ and normalized to ACTINB band intensity in the same sample.
(PDF)

Figure S5 **PKR mRNA is not induced in DENV-2 infected cells.** HuH-7 cells were infected with DENV-2 at MOI = 1 and harvested at various times post-infection. (A) Western blot analysis of G3BP1, G3BP2, CAPRIN1, viral capsid protein and PKR during the course of infection. (B) Quantitative real-time RT-PCR analysis of PKR mRNA induction normalized to intracellular GAPDH mRNA and uninfected control cells. Results are presented as mean ± SEM of three independent experiments.
(PDF)

Figure S6 **G3BP1 colocalizes with DENV-2 RNAs in infected cells.** HuH-7 cells were infected with DENV-2 at MOI = 1 for 24 h and stained by *in situ* hybridization combined with indirect immunofluorescence. DENV-2 genomic RNA was detected using a Alexa fluor-594 labeled antisense RNA probe complementary to the 5′ end of the genome (FISH DENV-2 5′UTR, red). The second antisense probe, complementary to the 3′ end of the genome thus detecting both gRNA and sfRNA, was labeled using a FITC-labeled antisense RNA probe (FISH DENV-2 3′UTR, green). Endogenous G3BP1 was detected using a Alexa fluor-633 labeled secondary antibody (pink). Nuclei were counterstained with DAPI. Note colocalization of G3BP1 and FISH DENV-2 3′UTR in cytoplasmic punctuated patterns that do not contain FISH DENV-2 5′UTR signal (white arrows), thus characteristic of sfRNA. Similar results were obtained when exchanging fluorophores on RNA probes (data not shown).
(PDF)

Figure S7 **Validation of differential quantitative real-time RT-PCR strategy to measure DENV-2 sfRNA.** (A and B) Overview of the differential real-time RT-PCR strategy designed to discriminate between DENV-2 gRNA and sfRNA. The DENV-2 NGC 3′UTR (A) contains conserved secondary structures SL-I to SL-V, DB1, DB2 and 3′SLA and SLB. The DENV-2 sfRNA (highlighted in grey) is derived from processing of the viral genome and is identical to the last 428 nucleotides of the DENV-2 3′UTR (the 3′UTR starts at the stop codon, UAG, indicated in red). Primer QG-For, annealing upstream of the stop codon, is designed to detect DENV-2 gRNA only. Primer QGSF-For, annealing downstream of SL-II, is designed to recognize both gRNA and sfRNA. The reverse primer QGSF-Rev is shared, leading to products of 309 and 184 nt, respectively. To calculate the amount of sfRNA (n(sfRNA)), absolute quantities of amplicons QG (n(G)) and QGSF (n(GSF)) are calculated against a standard curve generated with serial dilutions of D2Rep-RNA, mimicking the full-length DENV-2 genome. n(sfRNA) is then inferred by subtracting n(GSF)-n(G). Primers sequences and position in DENV-2 NGC sequence are indicated in (B). (C) Amplification efficiency does not differ significantly between primer pairs QG and QGSF. Serial dilutions of full-length D2Rep RNA were reverse transcribed and used in the assay described above. For each primer pair, results from three independent experiments were plotted as mean ± SEM of CT value obtained for increasing template concentrations. Equation of linear regressions and associated coefficient of determination (R^2^) are indicated. (D and E) The differential quantification method can discriminate between in-vitro gRNA/sfRNA ratios. Varying amounts of DENV-2 3′UTR, mimicking the sfRNA were mixed with a constant amount of full-length D2Rep-RNA (2.5fmoles, in the linear range of the assay as previously determined). Samples were reverse transcribed and ratios of sfRNA/gRNA determined using the assay (backcalculated, grey bars, D). The correlation between input ratio and ratio calculated by differential real-time RT-PCR (E) was robust for sfRNA/gRNA ratios greater than 1∶1. (F and G) Quantification of sfRNA/gRNA in infected samples. Total RNA was extracted from DENV-2 NGC virions (expected to contain exclusively gRNA) (bars in panel F indicate range of two independent determinations) and from HuH-7 cells infected for 24 h with DENV-2 NGC (F). Quantification shows no detectable sfRNA in virions. sfRNA and gRNA were quantified over the course of infection of 1×10^5^ HuH-7 cells, showing a 5–10-fold excess of sfRNA over gRNA at all tested time points (G).
(PDF)

Figure S8 **G3BP1, G3BP2 and CAPRIN1 immunoprecipitate DENV-2 gRNA and sfRNA from infected cells.** HuH-7 cells were infected with DENV-2 at MOI = 1 for 24 h and binding of host RBPs to viral and cellular RNAs was analyzed by RNA immunoprecipitation (RIP). Pellet fractions from IP with control anti-IgG, anti-G3BP1, anti-G3BP2 or anti-CAPRIN1 antibodies were analyzed for DENV-2 gRNA, DENV-2 sfRNA and cellular transcript c-Myc mRNA by real-time quantitative RT-PCR. Results are presented as mean ± SEM of the fold change (calculated by ddCT) of aforementioned RNAs over GAPDH mRNA in the pellet fraction, normalized to same value for control α-IgG IP from two independent experiments.
(PDF)

Figure S9 **Interaction of sfRNA with G3BP1, G3BP2 and CAPRIN1 is not conserved among flaviviruses.** (A to C) Selected flaviviral 3′UTR sequences (A) were aligned using CLUSTALW (B) and used to construct a CLUSTALW phylogenetic tree (C). Complementary sequences of structural elements are highlighted (dark blue: lower stem of SL-II, light blue: higher stem of SL-II, pink: pseudoknot PKSL-II). Secondary structure prediction of the variable region (SL-I to SL-V) of DENV-2 3′UTR and mutant dSLII-ST4 was generated using M-fold. (D and E) Interaction of RNAs spanning selected flaviviral 3′UTRs with G3BP1, G3BP2 and CAPRIN1. 3′UTR sequences of selected flaviviruses were used in tobramycin RNA affinity chromatography experiments as described in Ward et al (4). Eluates were probed for G3BP1, G3BP2 and CAPRIN1. Streptavidin, which specifically binds to the aptamer sequence common to all constructs, was used as a control for pulldown efficiency (D). To confirm these results in the setting of infection, HuH-7 cells were infected by YFV-17D for 24 h and YFV-17D gRNA, YFV-17D sfRNA and cellular transcript c-Myc mRNA were detected in G3BP1 immunoprecipitates by quantitative real-time RT-PCR.
(PDF)

Figure S10 **DENV-2 3′UTR downregulates PKR protein expression.** Effect of ectopic expression of DENV-2 3′UTR on PKR expression. As in figure 6G to 6I, HuH-7 cells were transfected with increasing concentrations of DENV-2 3′UTR or DENV-2 3′UTR YFSLE for 4 h and treated with 100 UI/ml IFN-β for 4 h. Intracellular DENV-2 3′UTR RNA levels were measured by quantitative real-time RT-PCR (A). Induction of PKR mRNA and PKR protein were measured by quantitative real-time RT-PCR (B) and western blot (C), respectively. Results are presented as mean ± SEM of one representative experiment in triplicate.
(PDF)

Figure S11 **Association of ISG mRNAs with polysomes is particularly sensitive to ectopic expression of DENV-2 3′UTR.** Polysome fractionation of 2.10^7^ HuH-7 cells transfected with 1.25(A), 5 (B) or 20 µg (C) of *in vitro* transcribed DENV-2 3′UTR or DENV-2 3′UTR YFSLE-ST4. The percentage of IFITM2, PKR, GAPDH or ELF2 mRNA was across fractions was determined by quantitative real-time PCR. Conditions A, B and C were run in independent experiments.
(PDF)

Figure S12 **dSLII-ST4 mutation does not affect translation or sfRNA formation in mutant replicons.** (A) Sequence and structure of D2Rep-dSLII-ST4 3′UTR. D2Rep-WT and D2Rep-dSLII-ST4 3′UTR sequences were aligned using ClustalW (A) Asterisks indicate identity. Gap corresponds to SL-II deletion and the mismatches show point mutations in SL-IV. (B and C) Effect of dSLII-ST4 mutation on translation of input RNAs and sfRNA formation. In vitro transcribed D2Rep-WT and D2Rep-dSLII-ST4 RNAs were co-electroporated in HuH-7 cells together with a control RNA expressing Firefly luciferase under the control of β-globin UTRs. sfRNA and gRNA levels were measured at 72 h post-electroporation for each reporter replicon by quantitative real-time PCR (B). Renilla luciferase activity was measured at 4 h post-electroporation and normalized to Firefly luciferase activity to control for electroporation efficiency (C). All results are expressed as mean ± SEM of three independent experiments, each comprising three independent electroporations for each condition.
(PDF)

Text S1 **This supplementary text includes supporting methods, supplementary figure legends and supplementary references.**
(DOCX)
