# Correction: G3BP1, G3BP2 and CAPRIN1 Are Required for Translation of Interferon Stimulated mRNAs and Are Targeted by a Dengue Virus Non-coding RNA

**DOI:** 10.1371/journal.ppat.1006295

**Published:** 2017-03-28

**Authors:** Katell Bidet, Dhivya Dadlani, Mariano A. Garcia-Blanco

In the course of subsequent studies of the mechanism for the dependency of ISG translation on G3BP1, G3BP2 and CAPRIN1, the authors noted an error in the cloning of one of the two control constructs—ELF2-Fluc. The Fluc open reading frame (ORF) for the ELF2-luc was derived from a different plasmid than the open reading frame for the three other Fluc constructs (the GAPDH-Fluc control, and the IFITM2-Fluc and PKR-Fluc) (see Fig 4 in the original article). The authors had originally sequenced all the constructs but neglected to notice that the ELF2-Fluc ORF was from a different Fluc source, which was slightly different for the other three constructs. Since the authors were using these constructs to test translation efficiency, there was concern that even slight differences in the ORF could alter the results shown in Fig 4 and quantified in Table 1 of the original article. An ELF2-Fluc with the identical Fluc ORF as the other three constructs in Fig 4 was reconstructed, a new HuH-7 cell lines expressing this new ELF2-Fluc was created, an IFITM2-Fluc construct was created, and the experiment was repeated. New figures were made to explain these changes, which is included here as S1 Fig. The reconstruction was carried out by Dr. Kuo-Chieh Liao.

Although the results with the IFITM2 construct remained qualitatively as originally reported, the results with the ELF2 construct are closer to results with the original GAPDH control construct, diminishing the differences in translation efficiency between reporters driven by ISG UTRs and those driven by non-ISG UTRs. It is important to note that the result in the corrected Fig 4 does not contradict the previous results, but it makes the differences between controls and experimental less dramatic.

The conclusions of this publication that remain valid are as follows:

1. G3BP1, G3BP2 and CAPRIN1 are novel mediators of the antiviral IFN response (Figure 1). Shown for antiviral response to DENV-2 and YFV(17D).
2. G3BP1, G3BP2 and CAPRIN1 are not required for accumulation of ISG mRNAs but are required for accumulation of ISG proteins (Figures 2 and S4). Shown for six ISGs (PKR, RIG-I, ISG15, IFITM2, STAT1, and MX1) vs no effect on two non-ISGs (GAPDH and ACTINB).
3. G3BP1, G3BP2 and CAPRIN1 are critical regulators of ISG mRNA translation (Figure 3). Data support that G12C are not required for bulk translation (measured by [35S] methionine incorporation and rRNA distribution in polysome gradients), but a subset of endogenous cellular mRNAs are sensitive to G12C knockdown (major shifts of polysome peak fractions for IFITM2 and PKR RNAs).
4. DENV-2 interferes with ISG mRNA translation (Figure 5). Data indicate that DENV-2 infection downregulates translation of ISG mRNAs (IFITM2 and PKR) but not that of two non-ISG mRNAs (ELF2 and GAPDH) and thus phenocopies what we observed with G12C knockdown.
5. G3BP1, G3BP2 and CAPRIN1 interact with DENV-2 noncoding sfRNA during infection (Figure 6)
6. DENV-2 sfRNA downregulates ISG mRNA translation (Figure 7 and S11). Data are consistent with results obtained in Figure 3 with G12C depletion and in Figure 5 with DENV-2 infection.
7. The G3BP1, G3BP2 and CAPRIN1-sfRNA interaction protects DENV-2 replicons from IFN-ß (Figure 8)

To properly correct the original article, the authors have made substantial changes to the following sections: Results, Discussion, and Methods.

All changes to the original article are detailed in a marked-up PDF included as S1 File. A clean Word document version of the revised article is included as S2 File. The corrected Fig 4 and Table 1 are provided here.

**Fig 4:**
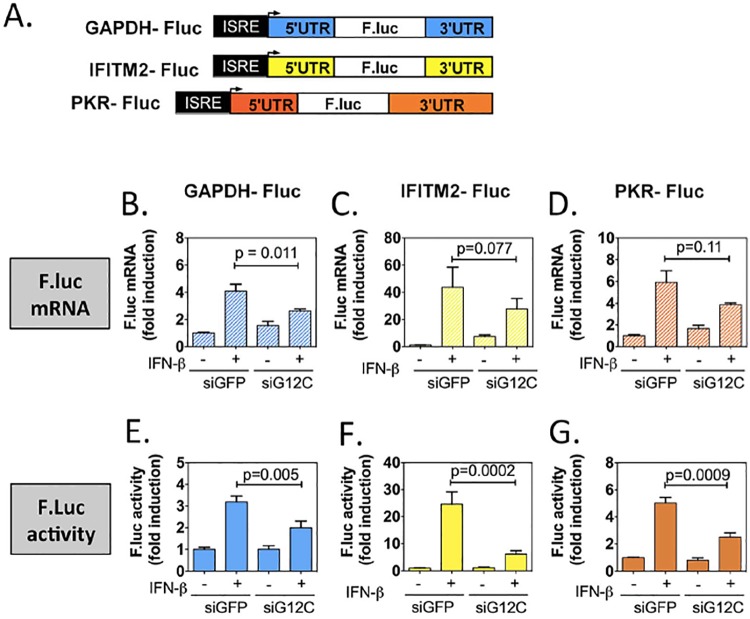
G3BP1, G3BP2 and CAPRIN1 depletion preferentially inhibits translation of reporters under the control of ISG UTRs. (A) Schematic representation of IFN-stimulated response element (ISRE)-driven firefly luciferase reporters under the control of GAPDH, IFITM2 or PKR UTRs. (B to G) HuH-7 cells stably transfected with the above constructs were treated with control siGFP or siG12C#1 siRNAs, induced with 1000UI/ml of IFN-β for 10h and firefly luciferase mRNA determined by quantitative real-time RT-PCR and normalized to GAPDH mRNA levels (B-D). Firefly luciferase protein levels were determined by measuring luciferase activity and normalized to total protein concentration (E-G). Both mRNA and protein activity are expressed as fold induction from control, untreated cells (siGFP, IFN-). Fluc measurements for the GAPDH-Fluc, IFITM2-Fluc and PKR-Fluc constructs were derived from 5 independent experiments in triplicate (n = 15). All Fluc mRNA levels were measured in two of these independent experiments (n = 6).

**Table 1:** Summary of mRNA induction, protein induction and translation efficiency for each Fluc reporter construct. Results presented in Fig 4 were compiled and for each construct, the relative translation efficiency was calculated as the ratio of the average firefly luciferase activity induction (normalized to siGFP, untreated cells set as 1) over to the average firefly luciferase mRNA induction (normalized to siGFP, untreated cells set as 1). The fold difference between siGFP + IFN and siG12C +IFN is shown in bold in the fourth column. The p-values for the differences in mRNA and Fluc induction between siGFP + IFN-β and siG12C + IFN-β conditions are indicated: ns, non significant; * p<0.05; ** p<0.01; ***p<0.005.

Construct	Biological process	siGFP + IFN-β	siG12C#1 + IFN-β
GAPDH-Fluc	mRNA induction	4.04 ± 0.49	2.63± 0.15 *
	Fluc induction	3.19 ± 0.26	1.91 ± 0.30 **
	Relative translation efficiency	0.78	0.73 **-1.07**
IFITM2-Fluc	mRNA induction	43.79 ± 14.5	27.79± 7.58 (ns)
	Fluc induction	24.55 ± 4.53	6.12 ± 1.32 ***
	Relative translation efficiency	0.56	0.22 **-2.54**
PKR-Fluc	mRNA induction	5.91 ± 1.06	3.85 ± 0.17 (ns)
	Fluc induction	5.01 ± 0.40	2.49 ± 0.32 ***
	Relative translation efficiency	0.85	0.65 **-1.3**

## Supporting information

S1 File Marked-up PDF detailing changes. (PDF)

S2 File Clean Word version of revised article. (DOCX)

S1 Fig G3BP1, G3BP2 and CAPRIN1 depletion decreases both mRNA and protein level from reconstructed ELF2-Fluc construct but diminishes only Fluc activity from IFITM2-Fluc. G3BP1, G3BP2 and CAPRIN1 depletion decreases both mRNA and protein level from new ELF2-Fluc construct but only diminishes Fluc activity from IFITM2-Fluc. (ISRE)-driven firefly luciferase reporters under the control of ELF2 (corrected version) or, IFITM2 UTRs. HuH-7 cells stably transfected with the above constructs were treated with control siGFP or siG12C#1 siRNAs, induced with 1000UI/ml of IFN-β for 10h and firefly luciferase mRNA and activity levels determined by quantitative real-time RT-PCR and normalized to HMBS and SDHA mRNA levels. Firefly luciferase protein levels were determined by measuring luciferase activity and normalized to total protein concentration (F-I). All results are expressed as fold induction from control, untreated cells. (Data of K C Liao and M A Garcia-Blanco)
(TIF)
