# Supplementary figures and images for: G3BP1, G3BP2 and CAPRIN1 Are Required for Translation of Interferon Stimulated mRNAs and Are Targeted by a Dengue Virus Non-coding RNA
Source: PLoS Pathog. 2014 Jul 3;10(7):e1004242. doi: 10.1371/journal.ppat.1004242 (PMC4081823; doi:10.1371/journal.ppat.1004242)

**Figure S1.**

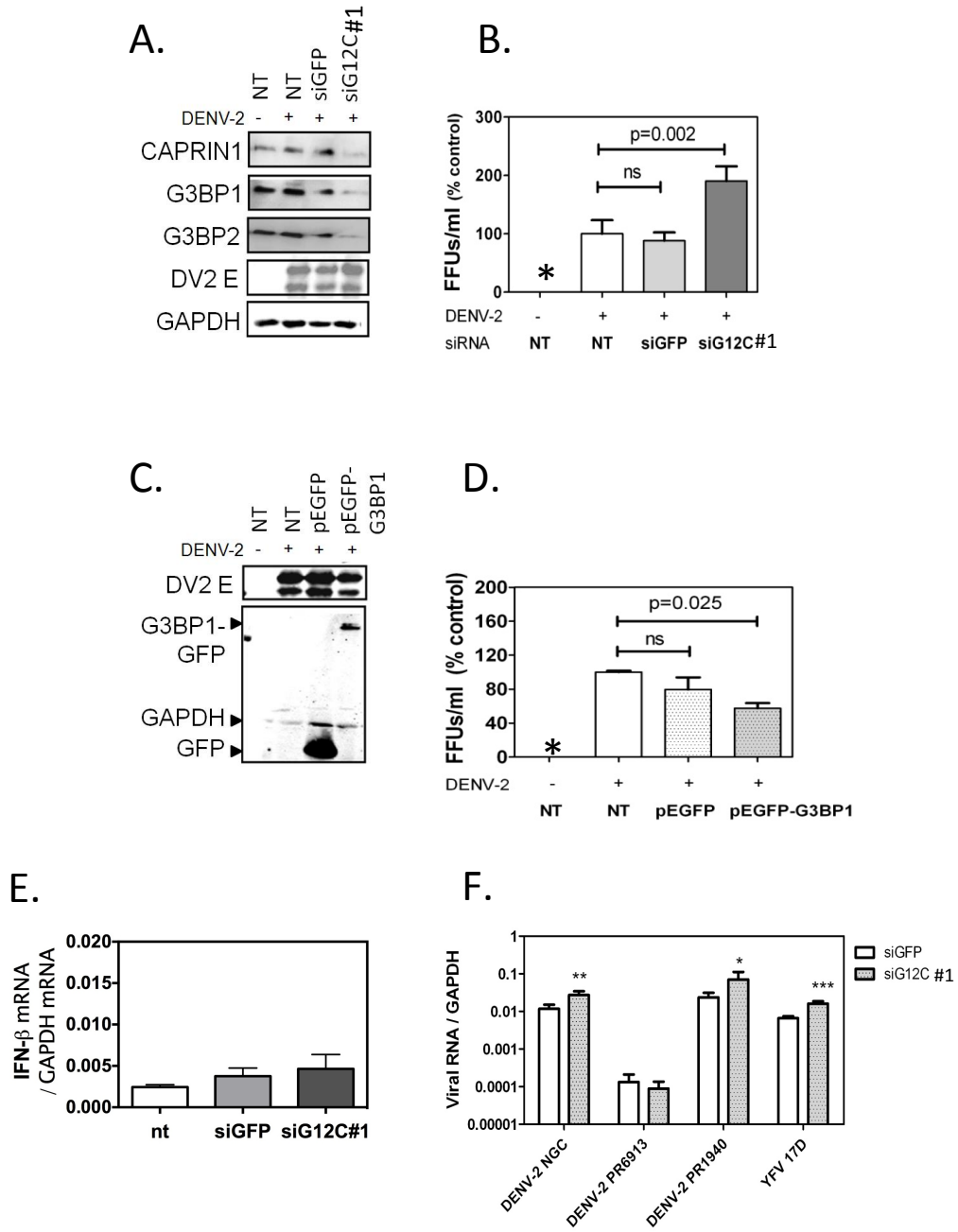

Supplement: Figure S1 — G3BP1, G3BP2 and CAPRIN1 have antiviral activity against DENV-2. (A–B) HuH-7 cells were left untreated (NT) or treated with control siRNA (siGFP) or siRNA targeting G3BP1, G3BP2 and CAPRIN1 (siG12C#1) and infected with DENV-2 NGC at MOI = 1 on day 5. Knockdown efficiency and viral protein expression were determined by western blot for G3BP1, G3BP2, CAPRIN1 and DENV-2 envelope (E) protein (A); infectious particle production was measured by focus forming assay at 24 h post infection and expressed as percent of the untreated, infected control from three independent experiments (B). (C–D) Effect of overexpression of G3BP1 on DENV-2 infection. HuH-7 cells were transfected with plasmids expressing either control (GFP) or GFP-tagged G3BP1 and infected with DENV-2 NGC at MOI = 1. Recombinant protein expression and viral protein expression were determined by western blot for DENV-2 E and GFP (C). Infectious particle production was measured by focus forming assay at 24 h post infection and expressed as percent untreated, infected control from 3 independent experiments (D). Asterisks indicate values below detection levels. (E) HuH-7 cells were left untreated (NT) or treated with control siRNA (siGFP) or siRNA targeting G3BP1, G3BP2 and CAPRIN1 (siG12C#1) twice over the course of four days. Lysates were collected on day 5 and levels of IFN-β mRNA measured by quantitative real-time RT-PCR and normalized to intracellular GAPDH mRNA levels. (F) G3BP1, G3BP2 and CAPRIN1 antiviral activity against a panel of flaviviruses. HuH-7 cells were treated with the indicated siRNAs (siGFP or siG12C) and infected with DENV-2 NGC, clinical isolates DENV-2 PR6913 and PR1940 or YFV-17D at MOI = 1. Viral RNA levels were measured at 24 h post-infection by quantitative real-time RT-PCR, using DENV-2 gRNA primer pair for DENV-2 NGC, DENV-2 PR6913 and DENV-2 PR1940 (100% primer sequence identity) and YFV-17D gRNA primer pair for YFV-17D. Results were expressed as fold induction compared to uninfected [file ppat.1004242.s001.pdf]

Figure S2.

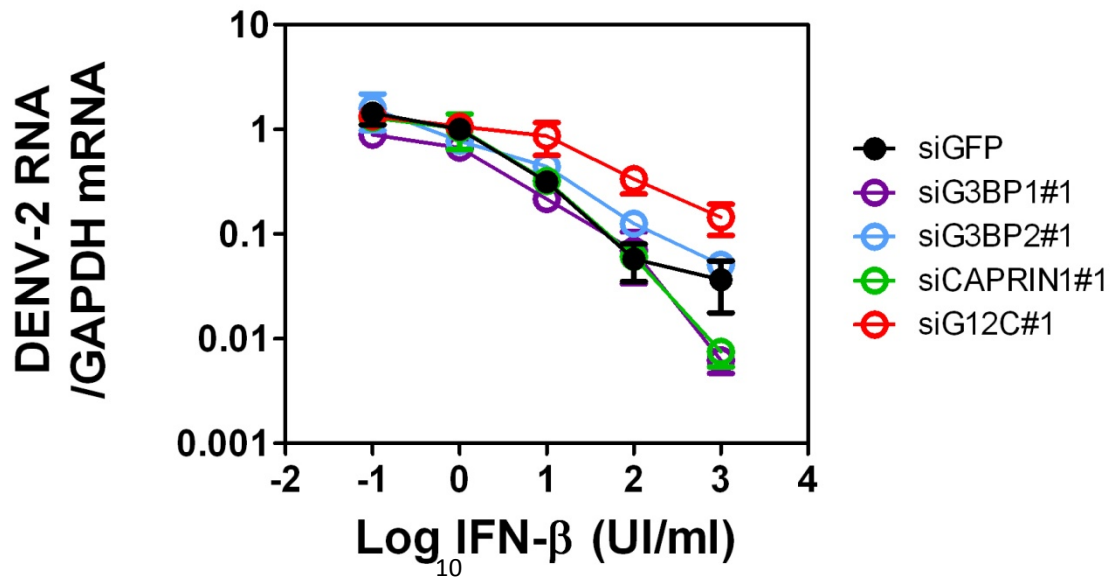

Supplement: Figure S2 — Individual depletion of G3BP1, G3BP2 or CAPRIN1 does not impair IFN-β mediated antiviral activity. HuH-7 cells were treated with control siRNA (siGFP, black), individual siRNAs targeting G3BP1, G3BP2 or CAPRIN1 (siG3BP1#1, siG3BP2#1, siCAPRIN1#1, purple, blue and green, respectively), or a pool of all three siRNAs (siG12C#1, red), pretreated with increasing concentrations of IFN-β and infected with DENV-2 at MOI = 1. Viral RNA levels were determined at 24 h post-infection by quantitative real-time RT-PCR and normalized to intracellular GAPDH mRNA levels. Results are presented as mean ± SEM of two independent experiments in duplicate. (PDF) [file ppat.1004242.s002.pdf]

**Figure S4.**

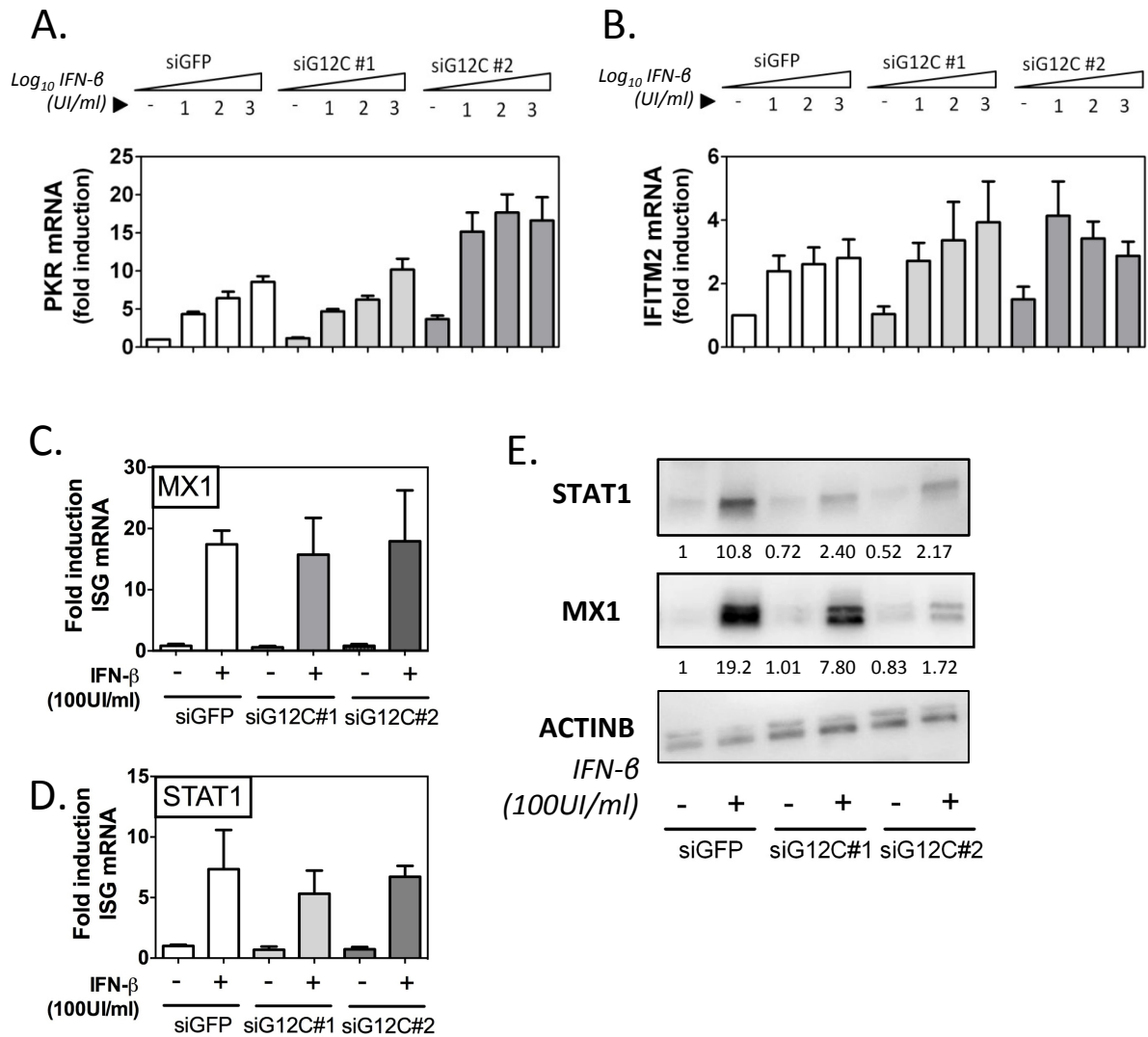

Supplement: Figure S4 — G3BP1, G3BP2 and CAPRIN1 are dispensable for ISG mRNA induction but required for accumulation of MX1 and STAT1 proteins. HuH-7 cells treated with siGFP, siG12C#1 or siG12C#2 were stimulated with the indicated concentration of IFN-β for 16 h and ISG mRNA and protein levels were determined. (A–B) IFITM2 and PKR mRNA induction upon treatment with 0, 10, 100 or 1000 UI/ml IFN-β was determined by quantitative real-time RT-PCR, normalized to intracellular levels of GAPDH mRNA and expressed as fold induction compared to control, untreated cells. (C–D) The same method was applied to MX1 and STAT1 mRNA induction upon treatment with 100 UI/ml IFN-β. (E) Protein levels of MX1, STAT1 and ACTINB were analyzed by western blot using HRP-conjugated secondary antibodies. Band intensity was determined by densitometry analysis using ImageJ and normalized to ACTINB band intensity in the same sample. (PDF) [file ppat.1004242.s004.pdf]

Figure S5.

A.

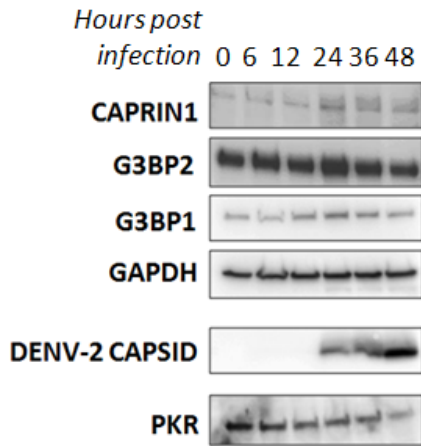

B.

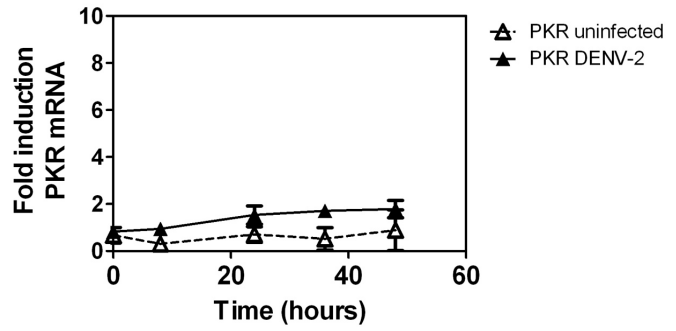

Supplement: Figure S5 — PKR mRNA is not induced in DENV-2 infected cells. HuH-7 cells were infected with DENV-2 at MOI = 1 and harvested at various times post-infection. (A) Western blot analysis of G3BP1, G3BP2, CAPRIN1, viral capsid protein and PKR during the course of infection. (B) Quantitative real-time RT-PCR analysis of PKR mRNA induction normalized to intracellular GAPDH mRNA and uninfected control cells. Results are presented as mean ± SEM of three independent experiments. (PDF) [file ppat.1004242.s005.pdf]

Figure S6.

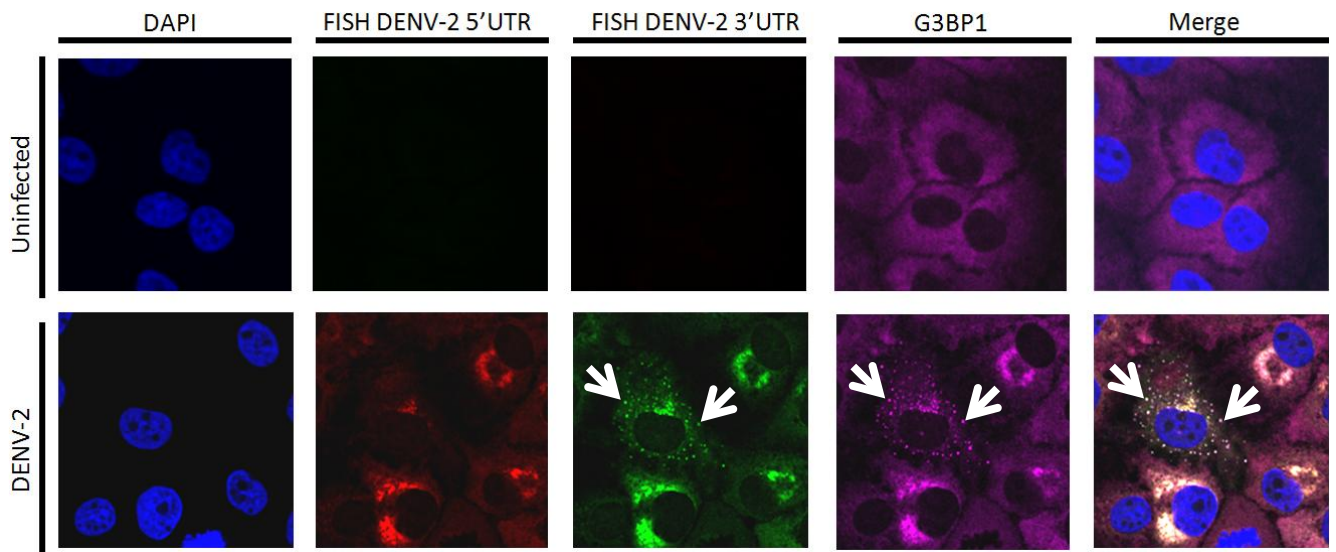

Supplement: Figure S6 — G3BP1 colocalizes with DENV-2 RNAs in infected cells. HuH-7 cells were infected with DENV-2 at MOI = 1 for 24 h and stained by in situ hybridization combined with indirect immunofluorescence. DENV-2 genomic RNA was detected using a Alexa fluor-594 labeled antisense RNA probe complementary to the 5′ end of the genome (FISH DENV-2 5′UTR, red). The second antisense probe, complementary to the 3′ end of the genome thus detecting both gRNA and sfRNA, was labeled using a FITC-labeled antisense RNA probe (FISH DENV-2 3′UTR, green). Endogenous G3BP1 was detected using a Alexa fluor-633 labeled secondary antibody (pink). Nuclei were counterstained with DAPI. Note colocalization of G3BP1 and FISH DENV-2 3′UTR in cytoplasmic punctuated patterns that do not contain FISH DENV-2 5′UTR signal (white arrows), thus characteristic of sfRNA. Similar results were obtained when exchanging fluorophores on RNA probes (data not shown). (PDF) [file ppat.1004242.s006.pdf]

Figure S8.

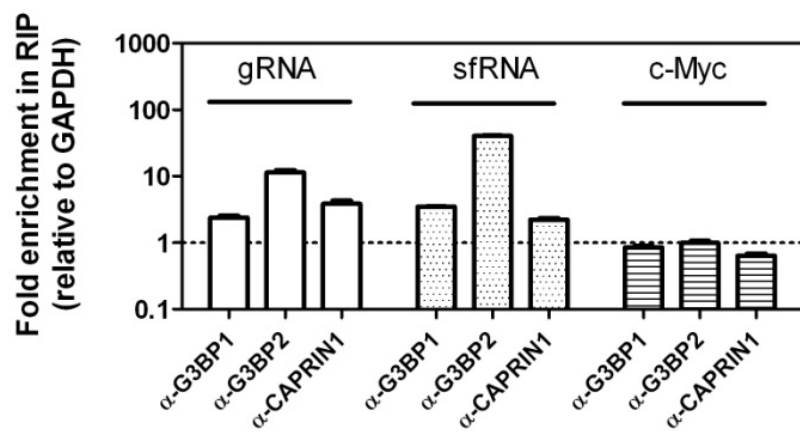

Supplement: Figure S8 — G3BP1, G3BP2 and CAPRIN1 immunoprecipitate DENV-2 gRNA and sfRNA from infected cells. HuH-7 cells were infected with DENV-2 at MOI = 1 for 24 h and binding of host RBPs to viral and cellular RNAs was analyzed by RNA immunoprecipitation (RIP). Pellet fractions from IP with control anti-IgG, anti-G3BP1, anti-G3BP2 or anti-CAPRIN1 antibodies were analyzed for DENV-2 gRNA, DENV-2 sfRNA and cellular transcript c-Myc mRNA by real-time quantitative RT-PCR. Results are presented as mean ± SEM of the fold change (calculated by ddCT) of aforementioned RNAs over GAPDH mRNA in the pellet fraction, normalized to same value for control α-IgG IP from two independent experiments. (PDF) [file ppat.1004242.s008.pdf]

Figure S9.

A.

|                |            |
|----------------|------------|
| DENV-2 NGC     | AF038403.1 |
| DENV-2 PR1940  | GQ398308.1 |
| DENV-2 PR5344  | GQ398283.1 |
| DENV-2 05K3295 | EU081177.1 |
| DENV-3 05K802  | EU081184.1 |
| DENV-3 05K4454 | EU081222.1 |
| KUNV           | AY274504.1 |
| YFV17D         | X03700.1   |

C.

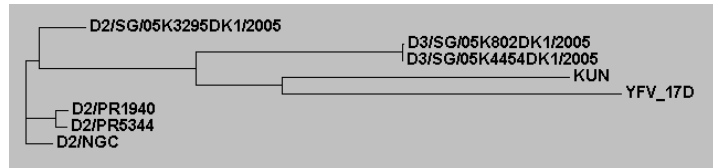

B.

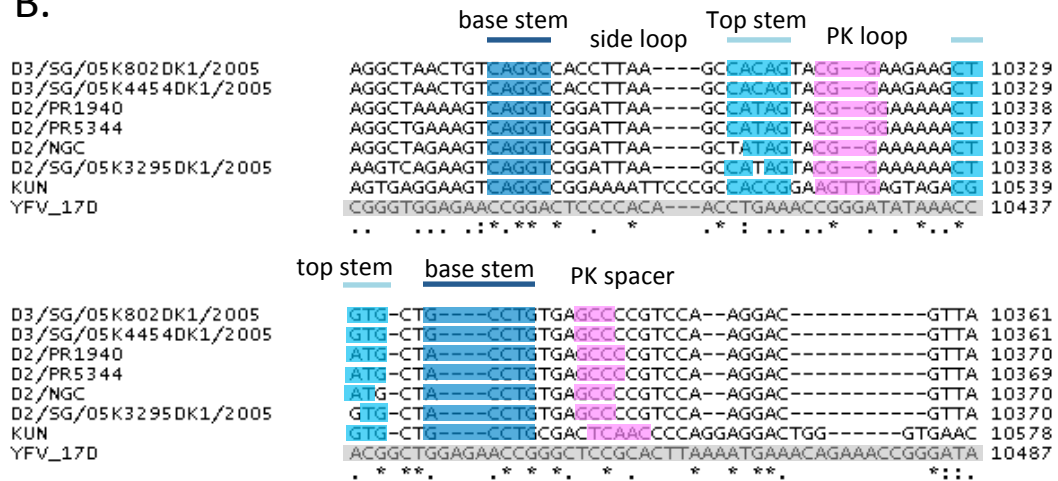

D.

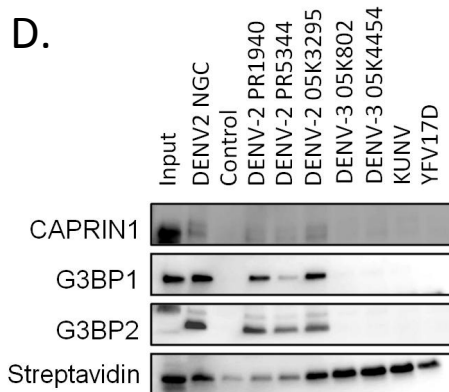

E.

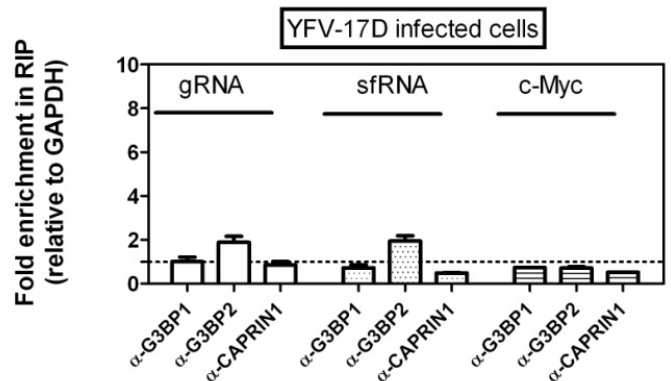

Supplement: Figure S9 — Interaction of sfRNA with G3BP1, G3BP2 and CAPRIN1 is not conserved among flaviviruses. (A to C) Selected flaviviral 3′UTR sequences (A) were aligned using CLUSTALW (B) and used to construct a CLUSTALW phylogenetic tree (C). Complementary sequences of structural elements are highlighted (dark blue: lower stem of SL-II, light blue: higher stem of SL-II, pink: pseudoknot PKSL-II). Secondary structure prediction of the variable region (SL-I to SL-V) of DENV-2 3′UTR and mutant dSLII-ST4 was generated using M-fold. (D and E) Interaction of RNAs spanning selected flaviviral 3′UTRs with G3BP1, G3BP2 and CAPRIN1. 3′UTR sequences of selected flaviviruses were used in tobramycin RNA affinity chromatography experiments as described in Ward et al (4). Eluates were probed for G3BP1, G3BP2 and CAPRIN1. Streptavidin, which specifically binds to the aptamer sequence common to all constructs, was used as a control for pulldown efficiency (D). To confirm these results in the setting of infection, HuH-7 cells were infected by YFV-17D for 24 h and YFV-17D gRNA, YFV-17D sfRNA and cellular transcript c-Myc mRNA were detected in G3BP1 immunoprecipitates by quantitative real-time RT-PCR. (PDF) [file ppat.1004242.s009.pdf]

**Figure S10.**

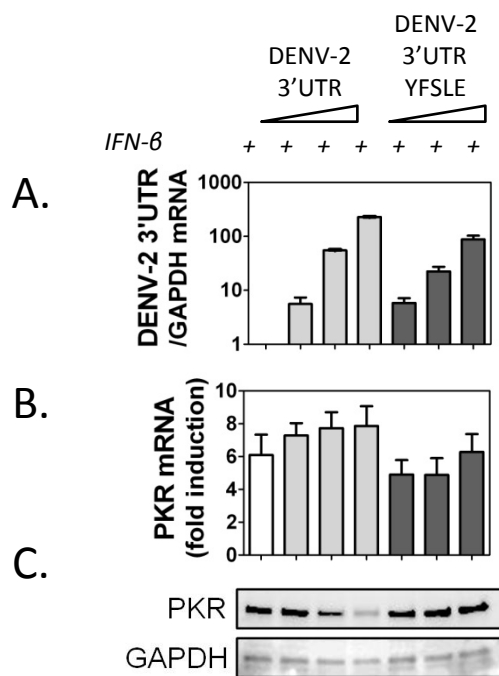

Supplement: Figure S10 — DENV-2 3′UTR downregulates PKR protein expression. Effect of ectopic expression of DENV-2 3′UTR on PKR expression. As in figure 6G to 6I, HuH-7 cells were transfected with increasing concentrations of DENV-2 3′UTR or DENV-2 3′UTR YFSLE for 4 h and treated with 100 UI/ml IFN-β for 4 h. Intracellular DENV-2 3′UTR RNA levels were measured by quantitative real-time RT-PCR (A). Induction of PKR mRNA and PKR protein were measured by quantitative real-time RT-PCR (B) and western blot (C), respectively. Results are presented as mean ± SEM of one representative experiment in triplicate. (PDF) [file ppat.1004242.s010.pdf]

**Figure S11.**

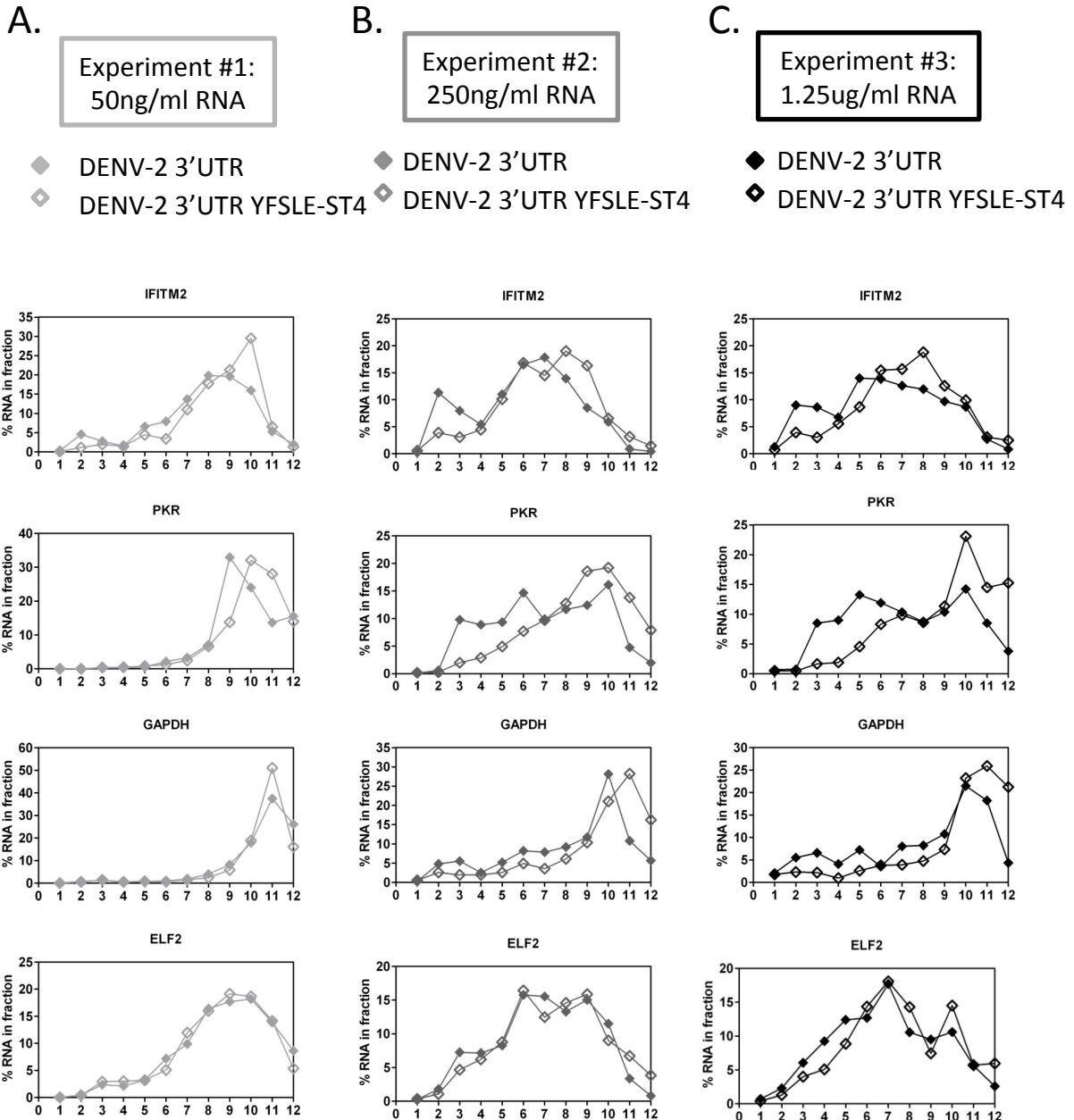

Supplement: Figure S11 — Association of ISG mRNAs with polysomes is particularly sensitive to ectopic expression of DENV-2 3′UTR. Polysome fractionation of 2.107 HuH-7 cells transfected with 1.25(A), 5 (B) or 20 µg (C) of in vitro transcribed DENV-2 3′UTR or DENV-2 3′UTR YFSLE-ST4. The percentage of IFITM2, PKR, GAPDH or ELF2 mRNA was across fractions was determined by quantitative real-time PCR. Conditions A, B and C were run in independent experiments. (PDF) [file ppat.1004242.s011.pdf]
