# Supplementary material for: G3BP1, G3BP2 and CAPRIN1 Are Required for Translation of Interferon Stimulated mRNAs and Are Targeted by a Dengue Virus Non-coding RNA
Source: PLoS Pathog. 2014 Jul 3;10(7):e1004242. doi: 10.1371/journal.ppat.1004242 (PMC4081823; doi:10.1371/journal.ppat.1004242)
Supplement: Figure S3 — IFN-β mediated antiviral activity against DENV-2 is not linked to stress granule (SG) formation. (A–C) DENV-2 inhibition by IFN-β is not accompanied by SG formation. Control or IFN-β treated HuH-7 cells were infected with DENV-2 at MOI = 1. The following IFN-β treatments were used: (#1) No IFN, (#2) 10 UI/ml 16 h prior to infection, (#3) 100 UI/ml 16 h prior to infection, (#4) 100 UI/ml 4 h after infection. Cells were fixed at 24 h post-infection and probed by indirect immunofluorescence for SG marker TIAL1 (TIAR, yellow) and DAPI (blue) (A). The percentage of SG-containing cells, defined as presenting more than 3 TIAR-containing cytoplasmic foci was determined manually (B – examples of positive cells are denoted by red arrows in panel A). The percentage of infected cells was determined by indirect immunofluorescence for dsRNA-containing replication complexes (C). Quantifications from one representative experiment (n>200 cells from one field) are shown. As described previously (ref), DENV-2 infection in control cells led to a slight induction of SG formation; pretreatment with increasing concentrations of IFN-β inhibiting DENV-2 replication did not affect the proportion of SG-containing cells nor did IFN-β treatment at 4 h post-infection, which did not affect viral replication. (D) The IFN-β response does not correlate with SG assembly. Cells pretreated with 100 UI/ml IFN-β and treated with SG inducers (50 mM sodium arsenite added to the media or 500 ng/ml polyI:C transfected with Lipofectamine 2000), or infected with DENV-2 were stained by indirect immunofluorescence for SG markers CAPRIN1 (green) and TIAR (red). The percentage of SG-containing cells (defined as cells with >3 CAPRIN1 and TIAR-containing cytoplasmic foci), was determined for >100 cells from one field and is indicated for each condition. (PDF) [file ppat.1004242.s003.pdf]

Figure S3.

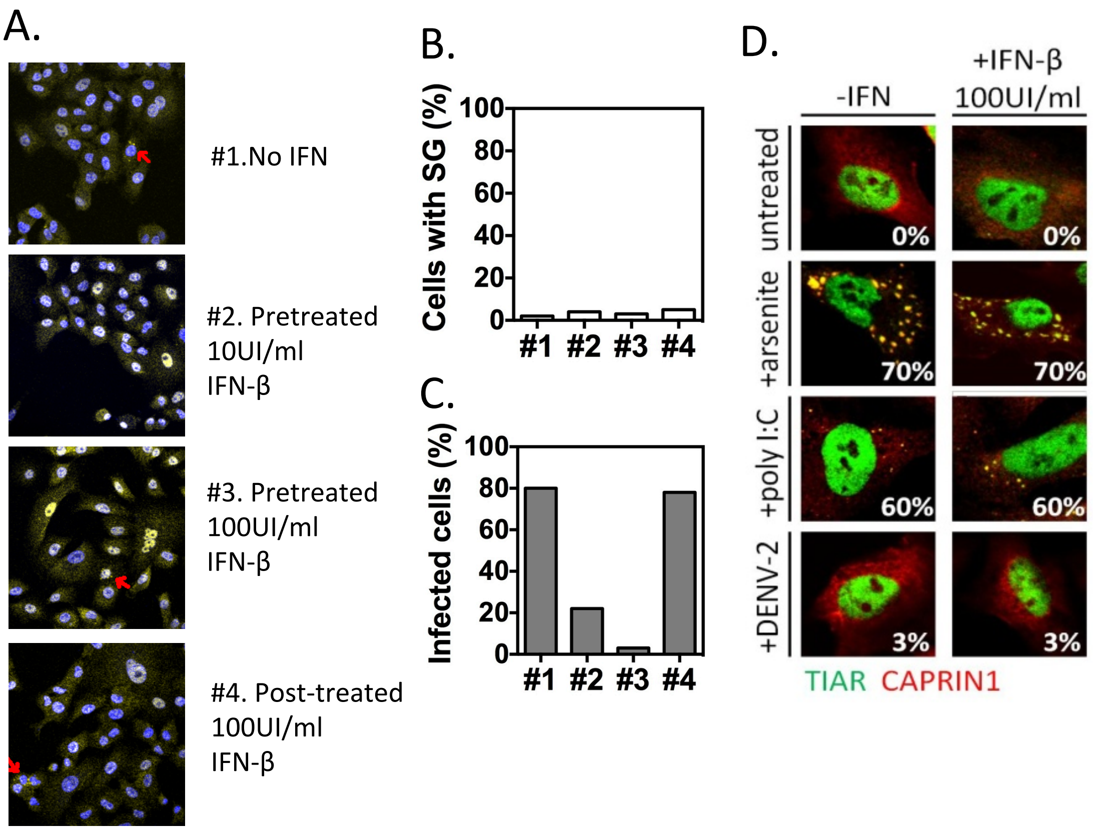

A.
#1.No IFN
#2. Pretreated 10UI/ml IFN-β
#3. Pretreated 100UI/ml IFN-β
#4. Post-treated 100UI/ml IFN-β
B.
Cells with SG (%)
100
80
60
40
20
0
#1 #2 #3 #4
C.
Infected cells (%)
100
80
60
40
20
0
#1 #2 #3 #4
D.
-IFN
+IFN-β 100UI/ml
untreated
+arsenite
+poly I:C
+DENV-2
0%
0%
70%
70%
60%
60%
3%
3%
TIAR CAPRIN1
