# Supplementary material for: G3BP1, G3BP2 and CAPRIN1 Are Required for Translation of Interferon Stimulated mRNAs and Are Targeted by a Dengue Virus Non-coding RNA
Source: PLoS Pathog. 2014 Jul 3;10(7):e1004242. doi: 10.1371/journal.ppat.1004242 (PMC4081823; doi:10.1371/journal.ppat.1004242)

Figure S7.

A.

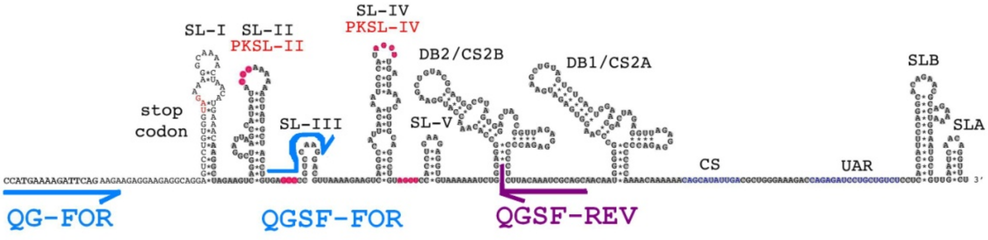

B.

Primer	Sequence	Position
QG_For	CCATGAAAAGATTCAGAAG	10201-10219
QGSF_For	GTGAGCCCCGTCCAAGG	10326-10342
QGSF_Rev	GCTGCGATTTGTAAGGG	10494-10510

C.

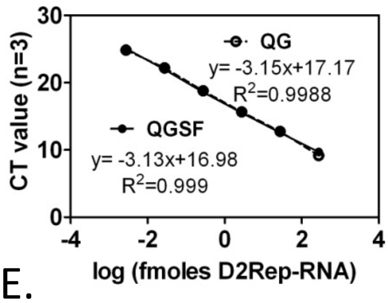

D.

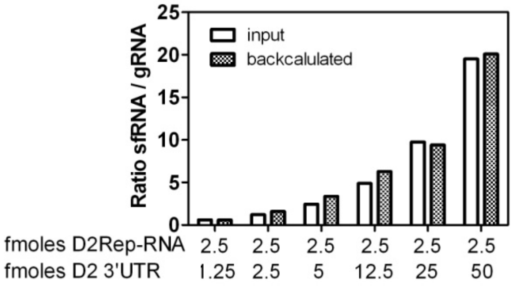

E.

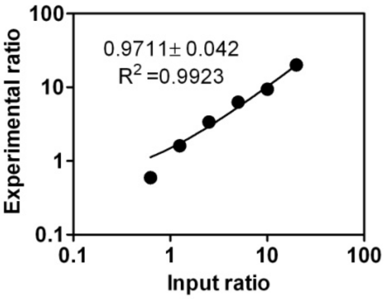

F.

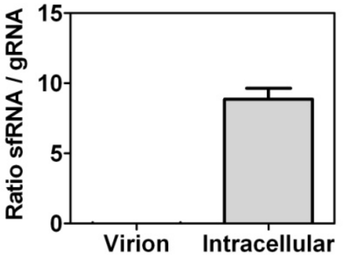

G.

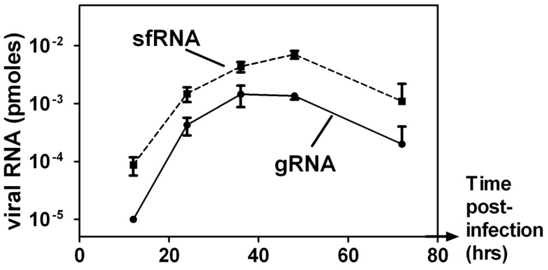

Supplement: Figure S7 — Validation of differential quantitative real-time RT-PCR strategy to measure DENV-2 sfRNA. (A and B) Overview of the differential real-time RT-PCR strategy designed to discriminate between DENV-2 gRNA and sfRNA. The DENV-2 NGC 3′UTR (A) contains conserved secondary structures SL-I to SL-V, DB1, DB2 and 3′SLA and SLB. The DENV-2 sfRNA (highlighted in grey) is derived from processing of the viral genome and is identical to the last 428 nucleotides of the DENV-2 3′UTR (the 3′UTR starts at the stop codon, UAG, indicated in red). Primer QG-For, annealing upstream of the stop codon, is designed to detect DENV-2 gRNA only. Primer QGSF-For, annealing downstream of SL-II, is designed to recognize both gRNA and sfRNA. The reverse primer QGSF-Rev is shared, leading to products of 309 and 184 nt, respectively. To calculate the amount of sfRNA (n(sfRNA)), absolute quantities of amplicons QG (n(G)) and QGSF (n(GSF)) are calculated against a standard curve generated with serial dilutions of D2Rep-RNA, mimicking the full-length DENV-2 genome. n(sfRNA) is then inferred by subtracting n(GSF)-n(G). Primers sequences and position in DENV-2 NGC sequence are indicated in (B). (C) Amplification efficiency does not differ significantly between primer pairs QG and QGSF. Serial dilutions of full-length D2Rep RNA were reverse transcribed and used in the assay described above. For each primer pair, results from three independent experiments were plotted as mean ± SEM of CT value obtained for increasing template concentrations. Equation of linear regressions and associated coefficient of determination (R2) are indicated. (D and E) The differential quantification method can discriminate between in-vitro gRNA/sfRNA ratios. Varying amounts of DENV-2 3′UTR, mimicking the sfRNA were mixed with a constant amount of full-length D2Rep-RNA (2.5fmoles, in the linear range of the assay as previously determined). Samples were reverse transcribed and ratios of sfRNA/gRNA determined using the assay (backcalc [file ppat.1004242.s007.pdf]
