# Supplementary material for: G3BP1, G3BP2 and CAPRIN1 Are Required for Translation of Interferon Stimulated mRNAs and Are Targeted by a Dengue Virus Non-coding RNA
Source: PLoS Pathog. 2014 Jul 3;10(7):e1004242. doi: 10.1371/journal.ppat.1004242 (PMC4081823; doi:10.1371/journal.ppat.1004242)

# Figure S12.

A.

```
DENV-2_16681_3'UTR         TAGAAAGCAAAACTAACATGAAACAAGGCTAGAAGTCAGGTCGGATTAAG
DENV-2_16881_dSLII/ST4     TAGAAAGCAAAACTAACATGAAACAAGGCTAGAAGTCAGG----------
                           ****************************************

DENV-2_16681_3'UTR         CCATAGTACGGAAAAAACTATGCTACCTGTGAGCCCCGTCCAAGGACGTT
DENV-2_16881_dSLII/ST4     ------------------------------GAGCCCCGTCCAAGGACGTT
                                                         ********************

DENV-2_16681_3'UTR         AAAAGAAGTCAGGCCATCATAAATGCCATAGCTTGAGTAAACTATGCAGC
DENV-2_16881_dSLII/ST4     AAAAGAAGTCAGGCCATACATAATGCCATAGCTTGAGTAAACTATGCAGC
                           *****************..::*****************************

DENV-2_16681_3'UTR         CTGTAGCTCCACCTGAGAAGGTGTAAAAAATCCGGGAGGCCACAAACCAT
DENV-2_16881_dSLII/ST4     CTGTAGCTCCACCTGAGAAGGTGTAAAAAATCCGGGAGGCCACAAACCAT
                           **************************************************

DENV-2_16681_3'UTR         GGAAGCTGTACGCATGGCGTAGTGGACTAGCGGTTAGAGGAGACCCCTCC
DENV-2_16881_dSLII/ST4     GGAAGCTGTACGCATGGCGTAGTGGACTAGCGGTTAGAGGAGACCCCTCC
                           **************************************************

DENV-2_16681_3'UTR         CTTACAAATCGCAGCAACAATGGGGGCCCAAGGCGAGATGAAGCTGTAGT
DENV-2_16881_dSLII/ST4     CTTACAAATCGCAGCAACAATGGGGGCCCAAGGCGAGATGAAGCTGTAGT
                           **************************************************

DENV-2_16681_3'UTR         CTCGCTGGAAGGACTAGAGGTTAGAGGAGACCCCCCCGAAACAAAAAACA
DENV-2_16881_dSLII/ST4     CTCGCTGGAAGGACTAGAGGTTAGAGGAGACCCCCCCGAAACAAAAAACA
                           **************************************************

DENV-2_16681_3'UTR         GCATATTGACGCTGGGAAAGACCAGAGATCCTGCTGTCTCCTCAGCATCA
DENV-2_16881_dSLII/ST4     GCATATTGACGCTGGGAAAGACCAGAGATCCTGCTGTCTCCTCAGCATCA
                           **************************************************

DENV-2_16681_3'UTR         TTCCAGGCACAGAACGCCAGAAAATGGAATGGTGCTGTTGAATCAACAGG
DENV-2_16881_dSLII/ST4     TTCCAGGCACAGAACGCCAGAAAATGGAATGGTGCTGTTGAATCAACAGG
                           **************************************************

DENV-2_16681_3'UTR         TTCT
DENV-2_16881_dSLII/ST4     TTCT
                           ****
```

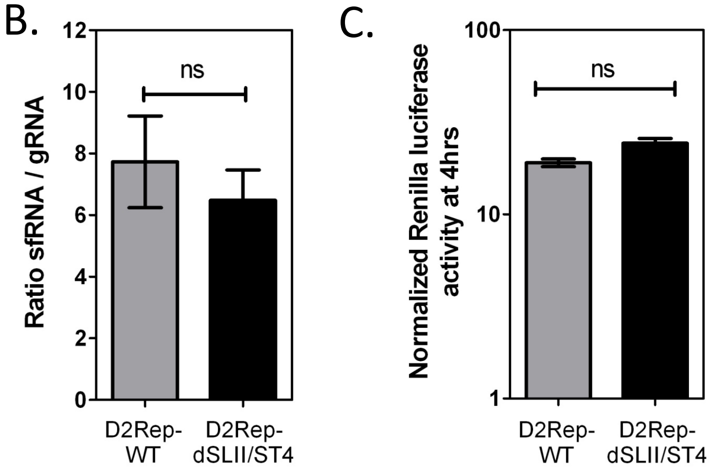

Supplement: Figure S12 — dSLII-ST4 mutation does not affect translation or sfRNA formation in mutant replicons. (A) Sequence and structure of D2Rep-dSLII-ST4 3′UTR. D2Rep-WT and D2Rep-dSLII-ST4 3′UTR sequences were aligned using ClustalW (A) Asterisks indicate identity. Gap corresponds to SL-II deletion and the mismatches show point mutations in SL-IV. (B and C) Effect of dSLII-ST4 mutation on translation of input RNAs and sfRNA formation. In vitro transcribed D2Rep-WT and D2Rep-dSLII-ST4 RNAs were co-electroporated in HuH-7 cells together with a control RNA expressing Firefly luciferase under the control of β-globin UTRs. sfRNA and gRNA levels were measured at 72 h post-electroporation for each reporter replicon by quantitative real-time PCR (B). Renilla luciferase activity was measured at 4 h post-electroporation and normalized to Firefly luciferase activity to control for electroporation efficiency (C). All results are expressed as mean ± SEM of three independent experiments, each comprising three independent electroporations for each condition. (PDF) [file ppat.1004242.s012.pdf]
