# Supplementary material for: G3BP1, G3BP2 and CAPRIN1 Are Required for Translation of Interferon Stimulated mRNAs and Are Targeted by a Dengue Virus Non-coding RNA
Source: PLoS Pathog. 2014 Jul 3;10(7):e1004242. doi: 10.1371/journal.ppat.1004242 (PMC4081823; doi:10.1371/journal.ppat.1004242)
Supplement: Text S1 — This supplementary text includes supporting methods, supplementary figure legends and supplementary references. (DOCX) [file ppat.1004242.s013.docx]

**SUPPLEMENTARY METHODS**

**Antibodies.**

Mouse anti-G3BP1 (BD Biosciences), rabbit anti-CAPRIN1 (a kind kift from G. Katsafanas and B. Moss, NIH), mouse anti-TIAR (BD Biosciences) and mouse anti-dsRNA J2 (English and Scientific consulting) antibodies were used for indirect immunofluorescence. Secondary Alexa-fluor conjugated (anti-mouse- 488, -594 or -633, anti-rabbit-488 or -594) (Invitrogen) were used as indicated. Mouse anti-ACTINB (Santa Cruz), anti-G3BP1 and anti-TIAR (BD Biosciences), and rabbit anti-G3BP2, anti-DDX6 (Bethyl), anti-CAPRIN1, anti-IFITM2 (Proteintech), anti-GFP and anti-MX1 (Abcam), anti-GAPDH, anti-PKR, anti-RIG-I, anti–ISG15 and anti-STAT1 (Cell Signaling) were used for western blot analysis. Secondary detection was routinely performed using HRP-conjugated secondary antibodies (Jackson Immunotech). If protein aboslute levels were to be quantified, fluorescent secondary antibodies anti-rabbit DyLight 800 and anti-mouse DyLight 680 (Pierce) were used in the Licor Odyssey system. DENV-2 was detected with mouse anti-envelope protein 4G2 or anti-capsid protein as previously described ([1](#_ENREF_1)). All RNA-immunoprecipitations were carried out using rabbit anti-KSRP, anti-G3BP1, anti-G3BP2 and anti-CAPRIN1 antibodies from Bethyl.

**siRNAs.**

The following siRNA duplexes were obtained from Integrated DNA Technologies and Invitrogen. The numbering indicate start position in target mRNA: control siGFP (5’ CGGCAAGCTGACCCTGAAGTTCAT 3’), siG3BP1 #1 (5’ GCCUGAGCCAGUAUUAGAATT 3’-813, coding sequence), siG3BP1#2 (5’ CCUUCUGGUGUGGAGAAAUTT 3’, 2056, 3’UTR), siG3BP2 #1 (5’ GGAAGAAUCCUCUCAUGAATT 3’, 867, coding sequence), siG3BP2 #2 (5’ CCGAGUGGCUCCUGAAUUUTT 3’, 3159, 3’UTR), siCAPRIN1 #1 (5’CCCUCCAGUUCAUUCUGAAUCUAGATT3’, 1413, coding sequence), siCAPRIN1 #2 (5’CAGCCCUCGAGUUAUUCAAUGAUAATT3’, 2690, 3’UTR).

**Overexpression studies.**

1.10^5^ HuH-7 cells (80% confluency) were transfected with 500ng CMV-driven expression vector pEGFP-N1 (Clontech) or the equivalent molar amount of pEGFP-G3BP1 (a gift from Dr Jamal Tazi, CNRS, France) using Lipofectamine 2000 (Invitrogen). Cells were infected 24h post transfection and assayed for DENV-2 replication after 24h.

**Quantification of DENV-2 infectivity.**

- **Quantification of percentage of DENV-2 infected cells.**

Quantification of percentage of infected cells was performed by indirect immunofluorescence for DENV-2 replication complexes. Briefly, cells were seeded on coverslips, infected with DENV-2 for 24h, fixed in 4% paraformaldehyde (PFA) and permeabilized using 0.1% Triton X-100. Replication complexes were detected using anti-dsRNA J2 antibody (1/200) (English and Scientific consulting) and Alexa fluor 594-conjugated anti-mouse secondary antibody (1/50). Nuclei were counterstained with DAPI. The percentage of infected cells was scored in a whole field (>200 cells) as the proportion of cells containing perinuclear dsRNA foci.

- **Quantification of DENV-2 RNA levels.**

DENV-2 RNA replication was assayed by quantitative real-time RT-PCR. 1-2μg total RNA was reversed transcribed in the presence of random primers using ImPromII (Promega), following manufacturer’s recommendations. Quantification using gRNA-specific primer pair 5’ GTG AGC CCC GTC CAA GG 3’ / 5’ GCT GCG ATT TGT AAG GG 3’ were performed using SYBR Green and the Biorad CFX96 detection system Absolute levels of RNA were calculated against a standard curve generated with full-length *in vitro* transcribed full length D2Rep RNA and normalized to intracellular GAPDH mRNA levels.

- **Quantification of infectious particle formation.**

DENV-2 infectious particle release was quantified using a focus forming assay as previously described ([1](#_ENREF_1)). Briefly, BHK-21 monolayers were infected with serial dilutions of experimental tissue culture supernatants, overlayed with 2% carboxymethylcellulose (CMC) and infection allowed to progress for 4 days. Cells were fixed in 4% formaldehyde and permeabilized using 0.1% Triton X-100. Viral foci were detected using the 3H5 monoclonal antibody to DENV E protein and anti-mouse DyLight 680 secondary antibody and visualized using the Odyssey infrared imaging system.

**Quantification of cellular mRNAs by quantitative real-time RT-PCR.**

1-2μg total RNA extracted using the RNAEasy kit (Qiagen) following manufacturer’s instructions was reverse transcribed with ImPromII RT (Promega). One tenth of each reaction was used for quantification of specific RNAs using the SYBR Green system (Biorad) and accompanied by a matching reaction quantifying GAPDH mRNA levels as a normalizer. Results were expressed as fold induction over untreated, unstimulated control cells normalized to GAPDH mRNA using the formula $2^{-ddCT}$ where

The following specific primer pairs were used: DENV-2 gRNA (5’ CCATGAAAAGATTCAGAAG 3’ / 5’ GCTGCGATTTGTAAGGG 3’); DENV-2 sfRNA (5’GTGAGCCCCGTCCAAGG 3’/ 5’ GCTGCGATT TGT AAGGG 3’); YFV-17D gRNA (5’ GGATGGAGAACCGGACTCC 3’ /5’ CGTCTTTCTACCACCACGTG 3’); YFV-17D sfRNA (5’ GCTAAGCTGTGAGGCAGTGC 3’/ 5’ CGTCTTTCTACCACCACGTG 3’); IFN-β (5’ CATTACCTGAAGGCCAAGGA 3’/ 5’ CAGCATCTGCTGGTTGAAGA 3’); EIF2AK2/PKR (5’ GCAACTCTTTAGTGACCAGC 3’/ 5’ CCGTCAATTCTGTGTTTTGC 3’); IFITM2 (5’ CGATCACCGCTGGTCACCATGAA 3’/ 5’ CCCGGTCACGTCGCCAACC 3’); DDX58/RIG-I (5’ GGACGTGGCAAAACAAATCAG 3’/ 5’ GCAATGTCAATGCCTTCATCA 3’); ISG15 (5’ GAGAGGCAGCGAACTCATCT 3’/ 5’ CTTCAGCTCTGACACCGACA 3’); MX1 (5’ TTCAGCACCTGATGGCCTATC 3’/ 5’ TGGATGATCAAAGGGATGTGG 3’); STAT1 (5’ CTAGTGGAGTGGAAGCGGAG 3’/ 5’ CACCACAAACGAGCTCTGAA 3’); F-Luc (5’ TCTGAGGAGCCTTCAGGATT 3’/ 5’ AGATGGAACCTCTTGGCAAC 3’); GRP78/BIP (5’ GATCATCAACGAGCCTACG 3’/ 5’ GCTGGTCAAAGTCTTCTC C 3’); GAPDH (5’ CATGTTCCAATATGATTCCACC 3’/ 5’ CTCCACGACGTACTCAGC 3’); ELF2 (5’ GGACAGTTCGTGTGGCAATGC 3’/ 5’ GACTACCTTTGGAGAGGTCGC 3’).

**Indirect immunofluorescence of stress granule formation.**

Cells at 50% confluency were seeded on coverslips and treated with 0.5mM sodium arsenite (Sigma), transfected with dsRNA (1mg/ml polyI:C, Sigma, using Lipofectamine 2000, Invitrogen, following manufacturer’s recommendations), or infected with DENV-2 for 1h. Cells were fixed in 4% paraformaldehyde, permeabilized in 0.1% Triton X-100 and probed successively with anti-CAPRIN1 (1/200) and mouse anti-TIAR (1/1000) antibodies followed by secondary Alexa fluor 488-conjugated anti-mouse and Alexa fluor-594 anti-mouse antibodies (1/50). Images were visualized on a Zeiss LSM610 confocal microscope. SG formation was scored manually as the percentage of cells containing >3 foci positive for both CAPRIN1 and TIAR (n>200 cells).

**[^35^S]methionine/cysteine metabolic labeling.**

Cells treated with siRNA twice over the course of 4 days were stimulated or not with 100UI/ml IFN-β for 3.5h. Media was replace with starvation media (cysteine and methionine-free DMEM, Invitrogen, supplemented with 10% FBS) to deplete intracellular levels of these amino acids. After 30min, ^35^S-labeled EasyTAG EXPRESS35S protein labeling mix (PerkinElmer) was added at a final concentration of 30μCi/ml and incubated 1h at 37°C. Cells were lysed in RIPA buffer (50mM Tris pH8.0, 150mM NaCl, 0.5% sodium deoxycholate, 1% NP-10, 0.5% Triton-X100) Whole cell lysates were separated on 4-15% SDS-PAGE gels and radioactivity incorporation revealed by exposure on a phosphorscreen for 16h. Readings were performed using a phosphorimager (BioRad).

**ISRE-luciferase constructs.**

The following reference sequences were used for the design of ISRE-luciferase constructs: IFITM2 NM_006435.2; PKR (EIF2AK2) NM_002759.3; GAPDH NM_002046.3; ELF2 NM_201999.2. Plasmids containing the IFN-stimulated response element (ISRE) and a minimal promoter followed by the 5’UTR and first 30 coding nucleotides of the indicated mRNA, a BamHI restriction site, a NotI restriction site (SbfI and NotI for ELF2), the last 30 coding nucleotides and 3’UTR of the indicated mRNA were custom synthesized by GenScript. The ISRE sequence, obtained from pHTS-ISRE (Biomyx) was modified to remove BamHI and XbaI restriction sites. ISRE-UTRs fragments were cloned into pcDNA3.1 between the AseI and XhoI site for IFITM2, NdeI and XhoI for ELF2 or AseI and XbaI for PKR and GAPDH and the firefly luciferase coding sequence (from pTNT-Fluc, a kind gift from Dr. Shelton Bradrick, Duke University, USA) was inserted between the BamHI and NotI restriction sites (SbfI and NotI for ELF2). Plasmids were transfected into HuH-7 cells using jetPrime (Polyplus transfection) and selected with 1500μg/ml G418 (Gibco) for between 3 and 6 passages before experiments were carried out.

**In-situ hybridization.**

Combined *in situ* hybridization-immunofluorescence protocol was adapted from Pijlman et al ([2](#_ENREF_2)). T7 promoter-driven templates for antisense RNA probes complementary to nt 1-393 (5’FISH) and to nt 10366-10723 (3’FISH) of the DENV-2 NGC genome were amplified by PCR using the following primer pairs: 5’FISH, 5’ AGTTGTTAGTCTACGTGGACC 3’ / 5’ TAGCGTAATACGACTCACTATAGGGCGTCTCCTGTTCAAGATGTTCAG 3’; 3’FISH, 5’ CGTTAAAAGAAGTCAGGCCATTAC 3’ / 5’ TAGCGTAATACGACTCACTATAGGAGAACCTGTTGATTCAACAGCACC 3’. RNA probes were *in vitro* transcribed using a T7 Megascript kit (Ambion) and labeled using a FITC ribonucleotide mix (Roche) or an Alexa fluor 594 FISH-TAG RNA lebeling kit (Invitrogen). G3BP1 was detected by indirect immunofluorecence using an Alexa fluor-633 anti-mouse secondary antibody. Slides were imaged using a Zeiss LSM710 confocal microscope and processed with the ZEN software.

**Bioinformatics analysis.**

Flavivirus 3’UTR sequences were aligned using the ClustalW server (www.ebi.ac.uk/Tools/msa/**clustalw**2/‎). The following reference sequences were used as input: DENV-2 strain NGC (AF038403.1), DENV-2 strain PR1940 (GQ398308.1), DENV-2 strain PR5344 (GQ398283.1), DENV-2 strain EDEN 05K3295 (EU081177.1), DENV-3 strain EDEN 05K802 (EU081184.1), DENV-3 strain EDEN 05K4454 (EU081222.1), WNV strain Kunjin (AY274504.1), YFV strain 17D (X03700.1).

Prediction of individual secondary structures was performed by M-Fold (**mfold**.rna.albany.edu/?q=**mfold**‎). Prediction of conserved RNA secondary structures was obtained from ALIfold (rna.tbi.univie.ac.at/cgi-bin/RNA**alifold**.cgi‎) using an alignment of the above flaviviral 3’UTRs as input and checked against published data ([2-4](#_ENREF_2)).

**DENV-2 3’UTR mutants used in RNA chromatography.**

DENV-2 3’UTR mutants were obtained in the indicated background (tobramycin RNA affinity chromatography RNA aptamer template ([4](#_ENREF_4)), DENV-2 3’UTR or pD2Rep) using Quikchange site-directed mutagenesis (Stratagene). Primers used for mutagenesis were the following: DENV-2 dSLII, 5’ CAAGGCTAGAAGTCAG**—**GGAGCCCCGTCCAAGGACG 3’/ 5’ CGTCCTTGGACGGGGCTC—CCTGACTTCTAGCCTTG (position of the deletion is indicated with hyphen); DENV-2 PKSLII, 5’CGGATTAAGCCATAGTA**G**G**C**AAAAAACTATGCTACC 3’/ 5’ GGTAGCATAGTTTTTT**G**C**C**TACTATGGCTTAATCCG 3’;. For transfection experiments, DENV-2 3’UTR was amplified by PCR to add a T7 promoter (T7-3UTR-For: 5’ TAGCGTAATACGACTCACTATAGGTAGAAGGCAAAACTAACATG 3’ / 3UTR-Rev: 5’ AGAACCTGTTGATTCAACAGCACC 3’), *in vitro* transcribed and purified using RNEasy (Qiagen) and transfected in cells using Lipofectamine RNAiMax (Invitrogen).

**DENV-2 replicons assay.**

DENV-2 replicon, which is based on the DENV-2 16681 strain, was kindly provided by Dr Eva Harris ([5](#_ENREF_5)). Mutant D2Rep-dSLII-ST4 was constructed with two rounds of site-directed mutagenesis using the Quikchange II XL kit (Stratagene) following manufacturer’s recommendation. In the first step, the full SL-II structure was deleted using primer pair DENV-2 dSLII described above; in the second step, four point mutations were introduced in SL-IV using the primer pair DENV-2 ST4, 5’ CGTTAAAAGAAGTCAGGCCAT**ACAT**AATGCCATAGCTTGAGTAAACTATGC 3’ / 5’ GCATAGTTTACTCAAGCTATGGCATT**ATGT**ATGGCCTGACTTCTTTTAACG 3’ (point mutations indicated in bold). D2Rep plasmids were linearized with XbaI, *in vitro* transcribed with the T7 Megascript kit (Ambion) with a molar ratio of 1.3:1 m7GpppA cap analog (NEB) to ATP, and purified using an RNEasy kit (Qiagen). Pools of 1x10^7^ HuH-7 or HuH-7.5 cells/cuvette were co-electroporated with 10μg of either D2Rep-WT or D2Rep-dSLII-ST4 RNA and 2.5μg of control TNT-Fluc RNA (a gift from Dr. Shelton Bradrick, Duke University Medical Center) in replicate cuvettes and delivered by a single pulse of 270V, 950μF using a BioRad GenPulser. Cells were lysed at the indicated times and assayed for replicon luciferase activity using the dual-luciferase kit (Promega) and normalized to the firefly luciferase internal control. Data points from 3 independent experiments (each comprising 2 or 3 independent electroporations for each condition) were analyzed by unpaired two-tailed t-test. Since no significant difference between any condition was observed at 4h (p>0.05), replicon activity for 24, 48 and 72h time-points (where the activity of the internal control fell to background levels and did not allow accurate normalization) were normalized to the Renilla luciferase activity at 4h and expressed as fold change in replicon activity.

**Tobramycin RNA affinity chromatography.**

Tobramycin RNA affinity chromatography of DENV-2 NGC 3’UTR and mutants has been described elsewhere ([4](#_ENREF_4)). Mutants DENV-2 dSLII and PKSLII were obtained by Quikchange (Stratagene) site-directed mutagenesis of the original construct. 3’UTRs of clinical isolates DENV-2 Puerto Rico strains PR1940, PR5344, Singapore strain 05K3295 and DENV-3 Singapore strain 05K802 and 05K4454 were amplified and cloned from virus stocks (kindly provided by Dr Eng Eong Ooi, Duke-NUS Graduate Medical Schol, Singapore) and fused to aptamer sequences by site overlapping PCR as described earlier using the following primers: for DENV-2, 5’ GATAGTCGCGGGCCGGGTATGTGCGTCTGGATCCTATTAGAAAGCAAAACTAACATG 3’ / 5’ AGAACCTGTTGATTCAAC 3’; for DENV-3, 5’ GATAGTCGCGGGCCGGGTATGTGCGTCTGGATCCTATAGAGTTCCTGGACTACATGCC 3’ / 5’ AGAACCTGTTGATTCAAC 3’ . KUNV 3’UTR was custom synthesized by GenScript using reference sequence AY274504.1 and fused to aptamer sequence using primer pair 5’ GATAGTCGCGGGCCGGGTATGTGCGTCTGGATCCTATTGTAAATACTTTGTTAATTG 3’ / 5’ AGATCCTGTGTTCTCGCACC 3’. RNA affinity chromatography was performed essentially as described with the exception that 20μg/ml recombinant streptavidin (Pierce), binding to the aptamer sequence, was added to cell lysates to serve as a recovery control. Eluates were analyzed by western blotting with specific RBP antibodies and HRP-conjugated anti-streptavidin antibody (Pierce).

**SUPPLEMENTARY FIGURE LEGENDS.**

**Figure S1. G3BP1, G3BP2 and CAPRIN1 have antiviral activity against DENV-2.**

(A-B) HuH-7 cells were left untreated (NT) or treated with control siRNA (siGFP) or siRNA targeting G3BP1, G3BP2 and CAPRIN1 (siG12C#1) and infected with DENV-2 NGC at MOI=1 on day 5. Knockdown efficiency and viral protein expression were determined by western blot for G3BP1, G3BP2, CAPRIN1 and DENV-2 envelope (E) protein (A); infectious particle production was measured by focus forming assay at 24h post infection and expressed as percent of the untreated, infected control from three independent experiments (B). *(C-D)* Effect of overexpression of G3BP1 on DENV-2 infection. HuH-7 cells were transfected with plasmids expressing either control (GFP) or GFP-tagged G3BP1 and infected with DENV-2 NGC at MOI=1. Recombinant protein expression and viral protein expression were determined by western blot for DENV-2 E and GFP (C). Infectious particle production was measured by focus forming assay at 24h post infection and expressed as percent untreated, infected control from 3 independent experiments (D). Asterisks indicate values below detection levels. *(E)* HuH-7 cells were left untreated (NT) or treated with control siRNA (siGFP) or siRNA targeting G3BP1, G3BP2 and CAPRIN1 (siG12C#1) twice over the course of four days. Lysates were collected on day 5 and levels of IFN-β mRNA measured by quantitative real-time RT-PCR and normalized to intracellular GAPDH mRNA levels. (F) G3BP1, G3BP2 and CAPRIN1 antiviral activity against a panel of flaviviruses. HuH-7 cells were treated with the indicated siRNAs (siGFP or siG12C) and infected with DENV-2 NGC, clinical isolates DENV-2 PR6913 and PR1940 or YFV-17D at MOI=1. Viral RNA levels were measured at 24h post-infection by quantitative real-time RT-PCR, using DENV-2 gRNA primer pair for DENV-2 NGC, DENV-2 PR6913 and DENV-2 PR1940 (100% primer sequence identity) and YFV-17D gRNA primer pair for YFV-17D. Results were expressed as fold induction compared to uninfected cells and normalized to intracellular GAPDH mRNA levels. All results are presented as mean ± SEM from 3 independent experiments and analyzed using an unpaired two-tailed Student’s t-test. *p<0.05 **p<0.01.

**Figure S2. Individual depletion of G3BP1, G3BP2 or CAPRIN1 does not impair IFN-β mediated antiviral activity.**

HuH-7 cells were treated with control siRNA (siGFP, black), individual siRNAs targeting G3BP1, G3BP2 or CAPRIN1 (siG3BP1#1, siG3BP2#1, siCAPRIN1#1, purple, blue and green, respectively), or a pool of all three siRNAs (siG12C#1, red), pretreated with increasing concentrations of IFN-β and infected with DENV-2 at MOI=1. Viral RNA levels were determined at 24h post-infection by quantitative real-time RT-PCR and normalized to intracellular GAPDH mRNA levels. Results are presented as mean ± SEM of two independent experiments in duplicate.

**Figure S3. IFN-β mediated antiviral activity against DENV-2 is not linked to stress granule (SG) formation.**

(A-C) DENV-2 inhibition by IFN-β is not accompanied by SG formation. Control or IFN-β treated HuH-7 cells were infected with DENV-2 at MOI=1. The following IFN-β treatments were used: (#1) No IFN, (#2) 10UI/ml 16h prior to infection, (#3) 100UI/ml 16h prior to infection, (#4) 100UI/ml 4h after infection. Cells were fixed at 24h post-infection and probed by indirect immunofluorescence for SG marker TIAL1 (TIAR, yellow) and DAPI (blue) (A). The percentage of SG-containing cells, defined as presenting more than 3 TIAR-containing cytoplasmic foci was determined manually (B – examples of positive cells are denoted by red arrows in panel A). The percentage of infected cells was determined by indirect immunofluorescence for dsRNA-containing replication complexes (C). Quantifications from one representative experiment (n>200 cells from one field) are shown. As described previously (ref), DENV-2 infection in control cells led to a slight induction of SG formation; pretreatment with increasing concentrations of IFN-β inhibiting DENV-2 replication did not affect the proportion of SG-containing cells nor did IFN-β treatment at 4h post-infection, which did not affect viral replication.

(D) The IFN-β response does not correlate with SG assembly. Cells pretreated with 100UI/ml IFN-β and treated with SG inducers (50mM sodium arsenite added to the media or 500ng/ml polyI:C transfected with Lipofectamine 2000), or infected with DENV-2 were stained by indirect immunofluorescence for SG markers CAPRIN1 (green) and TIAR (red). The percentage of SG-containing cells (defined as cells with >3 CAPRIN1 and TIAR-containing cytoplasmic foci), was determined for >100 cells from one field and is indicated for each condition.

**Figure S4. G3BP1, G3BP2 and CAPRIN1 are dispensable for ISG mRNA induction but required for accumulation of MX1 and STAT1 proteins.**

HuH-7 cells treated with siGFP, siG12C#1 or siG12C#2 were stimulated with the indicated concentration of IFN-β for 16h and ISG mRNA and protein levels were determined. (A-B) IFITM2 and PKR mRNA induction upon treatment with 0, 10, 100 or 1000 UI/ml IFN-β was determined by quantitative real-time RT-PCR, normalized to intracellular levels of GAPDH mRNA and expressed as fold induction compared to control, untreated cells. (C-D) The same method was applied to MX1 and STAT1 mRNA induction upon treatment with 100 UI/ml IFN-β. (E) Protein levels of MX1, STAT1 and ACTINB were analyzed by western blot using HRP-conjugated secondary antibodies. Band intensity was determined by densitometry analysis using ImageJ and normalized to ACTINB band intensity in the same sample.

**Figure S5. PKR mRNA is not induced in DENV-2 infected cells.**

HuH-7 cells were infected with DENV-2 at MOI=1 and harvested at various times post-infection.

(A) Western blot analysis of G3BP1, G3BP2, CAPRIN1, viral capsid protein and PKR during the course of infection.

(B) Quantitative real-time RT-PCR analysis of PKR mRNA induction normalized to intracellular GAPDH mRNA and uninfected control cells. Results are presented as mean ± SEM of three independent experiments.

**Figure S6. G3BP1 colocalizes with DENV-2 RNAs in infected cells.**

HuH-7 cells were infected with DENV-2 at MOI=1 for 24h and stained by *in situ* hybridization combined with indirect immunofluorescence. DENV-2 genomic RNA was detected using a Alexa fluor-594 labeled antisense RNA probe complementary to the 5’ end of the genome (FISH DENV-2 5’UTR, red). The second antisense probe, complementary to the 3’ end of the genome thus detecting both gRNA and sfRNA, was labeled using a FITC-labeled antisense RNA probe (FISH DENV-2 3’UTR, green). Endogenous G3BP1 was detected using a Alexa fluor-633 labeled secondary antibody (pink). Nuclei were counterstained with DAPI. Note colocalization of G3BP1 and FISH DENV-2 3’UTR in cytoplasmic punctuated patterns that do not contain FISH DENV-2 5’UTR signal (white arrows), thus characteristic of sfRNA. Similar results were obtained when exchanging fluorophores on RNA probes (data not shown).

**Figure S7. Validation of differential quantitative real-time RT-PCR strategy to measure DENV-2 sfRNA.**

(A and B) Overview of the differential real-time RT-PCR strategy designed to discriminate between DENV-2 gRNA and sfRNA. The DENV-2 NGC 3’UTR (A) contains conserved secondary structures SL-I to SL-V, DB1, DB2 and 3’SLA and SLB. The DENV-2 sfRNA (highlighted in grey) is derived from processing of the viral genome and is identical to the last 428 nucleotides of the DENV-2 3’UTR (the 3’UTR starts at the stop codon, UAG, indicated in red). Primer QG-For, annealing upstream of the stop codon, is designed to detect DENV-2 gRNA only. Primer QGSF-For, annealing downstream of SL-II, is designed to recognize both gRNA and sfRNA. The reverse primer QGSF-Rev is shared, leading to products of 309 and 184nt, respectively. To calculate the amount of sfRNA (n(sfRNA)), absolute quantities of amplicons QG (n(G)) and QGSF (n(GSF)) are calculated against a standard curve generated with serial dilutions of D2Rep-RNA, mimicking the full-length DENV-2 genome. n(sfRNA) is then inferred by subtracting n(GSF)-n(G). Primers sequences and position in DENV-2 NGC sequence are indicated in (B).

*(C) Amplification efficiency does not differ significantly between primer pairs QG and QGSF.* Serial dilutions of full-length D2Rep RNA were reverse transcribed and used in the assay described above. For each primer pair, results from three independent experiments were plotted as mean ± SEM of CT value obtained for increasing template concentrations. Equation of linear regressions and associated coefficient of determination (R^2^) are indicated.

(D and E) The differential quantification method can discriminate between in-vitro gRNA/sfRNA ratios. Varying amounts of DENV-2 3’UTR, mimicking the sfRNA were mixed with a constant amount of full-length D2Rep-RNA (2.5fmoles, in the linear range of the assay as previously determined). Samples were reverse transcribed and ratios of sfRNA/gRNA determined using the assay (backcalculated, grey bars, D). The correlation between input ratio and ratio calculated by differential real-time RT-PCR (E) was robust for sfRNA/gRNA ratios greater than 1:1.

(F and G) Quantification of sfRNA/gRNA in infected samples. Total RNA was extracted from DENV-2 NGC virions (expected to contain exclusively gRNA) (bars in panel F indicate range of two independent determinations) and from HuH-7 cells infected for 24h with DENV-2 NGC (F). Quantification show no detectable sfRNA in virions. sfRNA and gRNA were quantified over the course of infection of 1 x 10^5^ HuH-7 cells, showing a 5-10-fold excess of sfRNA over gRNA at all tested time points (G).

**Figure S8. G3BP1, G3BP2 and CAPRIN1 immunoprecipitate DENV-2 gRNA and sfRNA from infected cells.**

HuH-7 cells were infected with DENV-2 at MOI=1 for 24h and binding of host RBPs to viral and cellular RNAs was analyzed by RNA immunoprecipitation (RIP). Pellet fractions from IP with control anti-IgG, anti-G3BP1, anti-G3BP2 or anti-CAPRIN1 antibodies were analyzed for DENV-2 gRNA, DENV-2 sfRNA and cellular transcript c-Myc mRNA by real-time quantitative RT-PCR. Results are presented as mean ± SEM of the fold change (calculated by ddCT) of aforementioned RNAs over GAPDH mRNA in the pellet fraction, normalized to same value for control α-IgG IP from two independent experiments.

**Figure S9. Interaction of sfRNA with G3BP1, G3BP2 and CAPRIN1 is not conserved among flaviviruses.**

(A to C) Selected flaviviral 3’UTR sequences (A) were aligned using CLUSTALW (B) and used to construct a CLUSTALW phylogenetic tree (C). Complementary sequences of structural elements are highlighted (dark blue: lower stem of SL-II, light blue: higher stem of SL-II, pink: pseudoknot PKSL-II). Secondary structure prediction of the variable region (SL-I to SL-V) of DENV-2 3’UTR and mutant dSLII-ST4 was generated using M-fold.

(D and E) Interaction of RNAs spanning selected flaviviral 3’UTRs with G3BP1, G3BP2 and CAPRIN1. 3’UTR sequences of selected flaviviruses were used in tobramycin RNA affinity chromatography experiments as described in Ward et al ([4](#_ENREF_4)). Eluates were probed for G3BP1, G3BP2 and CAPRIN1. Streptavidin, which specifically binds to the aptamer sequence common to all constructs, was used as a control for pulldown efficiency (D). To confirm these results in the setting of infection, HuH-7 cells were infected by YFV-17D for 24h and YFV-17D gRNA, YFV-17D sfRNA and cellular transcript c-Myc mRNA were detected in G3BP1 immunoprecipitates by quantitative real-time RT-PCR.

**Figure S10. DENV-2 3’UTR downregulates PKR protein expression.**

Effect of ectopic expression of DENV-2 3’UTR on PKR expression. As in figure 6G to 6I, HuH-7 cells were transfected with increasing concentrations of DENV-2 3’UTR or DENV-2 3’UTR YFSLE for 4h and treated with 100UI/ml IFN-β for 4h. Intracellular DENV-2 3’UTR RNA levels were measured by quantitative real-time RT-PCR (A). Induction of PKR mRNA and PKR protein were measured by quantitative real-time RT-PCR (B) and western blot (C), respectively. Results are presented as mean ± SEM of one representative experiment in triplicate.

**Figure S11. Association of ISG mRNAs with polysomes is particularly sensitive to ectopic expression of DENV-2 3’UTR.**

Polysome fractionation of 2.10^7^ HuH-7 cells transfected with 1.25(A), 5 (B) or 20µg (C) of *in vitro* transcribed DENV-2 3’UTR or DENV-2 3’UTR YFSLE-ST4. The percentage of IFITM2, PKR, GAPDH or ELF2 mRNA was across fractions was determined by quantitative real-time PCR. Conditions A, B and C were run in independent experiments.

**Figure S12. dSLII-ST4 mutation does not affect translation or sfRNA formation in mutant replicons.**

(A) Sequence and structure of D2Rep-dSLII-ST4 3’UTR. D2Rep-WT and D2Rep-dSLII-ST4 3’UTR sequences were aligned using ClustalW (A) Asterisks indicate identity. Gap corresponds to SL-II deletion and the mismatches show point mutations in SL-IV. .

(B and C) Effect of dSLII-ST4 mutation on translation of input RNAs and sfRNA formation. In vitro transcribed D2Rep-WT and D2Rep-dSLII-ST4 RNAs were co-electroporated in HuH-7 cells together with a control RNA expressing Firefly luciferase under the control of β-globin UTRs. sfRNA and gRNA levels were measured at 72h post-electroporation for each reporter replicon by quantitative real-time PCR (B). Renilla luciferase activity was measured at 4h post-electroporation and normalized to Firefly luciferase activity to control for electroporation efficiency (C). All results are expressed as mean ± SEM of three independent experiments, each comprising three independent electroporations for each condition.

**Supplementary references**

1. Sessions OM*, et al.* (2009) Discovery of insect and human dengue virus host factors. (Translated from eng) *Nature* 458(7241):1047-1050 (in eng).

2. Pijlman GP*, et al.* (2008) A highly structured, nuclease-resistant, noncoding RNA produced by flaviviruses is required for pathogenicity. (Translated from eng) *Cell Host Microbe* 4(6):579-591 (in eng).

3. Alvarez DE, De Lella Ezcurra AL, Fucito S, & Gamarnik AV (2005) Role of RNA structures present at the 3'UTR of dengue virus on translation, RNA synthesis, and viral replication. (Translated from eng) *Virology* 339(2):200-212 (in eng).

4. Ward AM*, et al.* (2011) Quantitative mass spectrometry of DENV-2 RNA-interacting proteins reveals that the DEAD-box RNA helicase DDX6 binds the DB1 and DB2 3' UTR structures. (Translated from eng) *RNA Biol* 8(6):1173-1186 (in eng).

5. Holden KL*, et al.* (2006) Inhibition of dengue virus translation and RNA synthesis by a morpholino oligomer targeted to the top of the terminal 3' stem-loop structure. (Translated from eng) *Virology* 344(2):439-452 (in eng).
