# Supplementary figures and images for: Correction: G3BP1, G3BP2 and CAPRIN1 Are Required for Translation of Interferon Stimulated mRNAs and Are Targeted by a Dengue Virus Non-coding RNA
Source: PLoS Pathog. 2017 Mar 28;13(3):e1006295. doi: 10.1371/journal.ppat.1006295 (PMC5370155; doi:10.1371/journal.ppat.1006295)

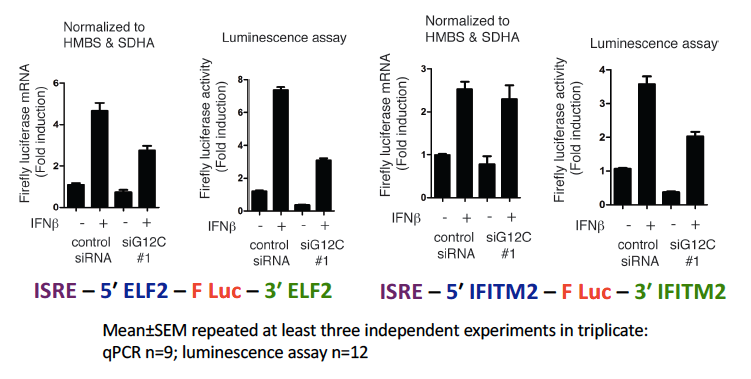

Supplement: S1 Fig — G3BP1, G3BP2 and CAPRIN1 depletion decreases both mRNA and protein level from new ELF2-Fluc construct but only diminishes Fluc activity from IFITM2-Fluc. (ISRE)-driven firefly luciferase reporters under the control of ELF2 (corrected version) or, IFITM2 UTRs. HuH-7 cells stably transfected with the above constructs were treated with control siGFP or siG12C#1 siRNAs, induced with 1000UI/ml of IFN-β for 10h and firefly luciferase mRNA and activity levels determined by quantitative real-time RT-PCR and normalized to HMBS and SDHA mRNA levels. Firefly luciferase protein levels were determined by measuring luciferase activity and normalized to total protein concentration (F-I). All results are expressed as fold induction from control, untreated cells. (Data of K C Liao and M A Garcia-Blanco) (TIF) [file ppat.1006295.s003.tif]
